# A novel test of flexible planning in relation to executive function and language in young children

**DOI:** 10.1098/rsos.192015

**Published:** 2020-04-15

**Authors:** Rachael Miller, Anna Frohnwieser, Ning Ding, Camille A. Troisi, Martina Schiestl, Romana Gruber, Alex H. Taylor, Sarah A. Jelbert, Markus Boeckle, Nicola S. Clayton

**Affiliations:** 1 Department of Psychology, University of Cambridge, Cambridge, UK; 2 School of Psychology, Auckland University, Auckland, New Zealand; 3 Department of Psychology and Psychodynamics, Karl Landsteiner University of Health Sciences, St Pölten, Austria; 4 School of Biological, Earth and Environmental Sciences, University College Cork, Cork, Ireland; 5 School of Psychological Science, University of Bristol, Bristol, UK

**Keywords:** flexible planning, executive function, language, child development, comparative cognition

## Abstract

In adult humans, decisions involving the choice and use of tools for future events typically require episodic foresight. Previous studies suggest some non-human species are capable of future planning; however, these experiments often cannot fully exclude alternative learning explanations. Here, we used a novel tool-use paradigm aiming to address these critiques to test flexible planning in 3- to 5-year-old children, in relation to executive function and language abilities. In the flexible planning task, children were not verbally cued during testing, single trials avoided consistent exposure to stimulus–reward relationships, and training trials provided experience of a predictable return of reward. Furthermore, unlike most standard developmental studies, we incorporated short delays before and after tool choice. The critical test choice included two tools with equal prior reward experience—each only functional in one apparatus. We tested executive function and language abilities using several standardized tasks. Our results echoed standard developmental research: 4- and 5-year-olds outperformed 3-year-olds on the flexible planning task, and 5-year-old children outperformed younger children in most executive function and language tasks. Flexible planning performance did not correlate with executive function and language performance. This paradigm could be used to investigate flexible planning in a tool-use context in non-human species.

## 1. Introduction

Future-oriented cognition is a critical part of human life. We spend a substantial amount of time on considering, planning and preparing for future scenarios [1]. Decisions involving the use of tools for a future event typically require episodic foresight, i.e. the ability to imagine future scenarios, and flexible planning outside of the present moment [2]. Children younger than 4 years appear to have difficulties in foreseeing future events and choosing correct items to address their future needs (for a review, see [3,4]).

Previous studies indicate that children's foresight ability undergoes important changes during preschool years [5]. Studies have shown that 4- and 5-year-olds were able to link past events from 15 min ago to deferred future problems and to select the correct tool in the present to solve the anticipated problems [6–8]. In contrast, 3-year-olds could only choose the appropriate tool to address needs that were of immediate concern, indicating that the delay between past event and future-directed action influenced children's performance. Subsequent research investigated children's performance with different lengths of delay and reported similar findings. Three-year-olds were only successful in choosing the correct tool to address their current needs but failed to select the correct tool to address their future needs [9–11]. Foresight is an important part of flexible planning, and although most research indicates that 4-year-olds pass flexible planning tests, Dickerson and colleagues found that this may be related to the type of task used [12]. In this study, the flexible planning task aimed to control for the association between a choice and its salience, by presenting children with a choice of two high-value objects after seeing a specific problem. Only one object could be used to solve the observed problem. Four-year-olds failed to pass the test, while 5- to 7-year-olds passed it. Hence, the authors suggested that 4-year-olds may use a simpler mechanism to solve prior flexible planning experimental tasks without necessarily possessing flexible planning ability. Specifically, positively associated objects may be chosen even in the absence of a possible future event [12].

Previous research that required a language component has suggested that failure in flexible planning may be attributed to a limited understanding of language [13]. Thus, non-linguistic behavioural-based paradigms have been widely adopted in developmental research [14]. Specifically, researchers have designed tasks to assess the ability to use past information to prepare current actions for a future problem in both young children and other animals [7,8,15]. For example, corvids (members of the crow family) have shown impressive flexible planning skills in caching tasks, for example in learning what to cache and what not to cache based on the foods available at the time of cache recovery [15–21]. Studies on corvids and other species have also indicated flexible planning behaviours beyond natural behavioural predispositions, i.e. outside of the caching context [22–27]. Recently, ravens were shown to be able to plan for future tool use and barter with delays up to 17 h [22].

However, some of the non-human studies face several critiques relating to possible learning explanations [28]. Namely, the use of multiple trials could reinforce the repeated exposure to the same stimulus–reward relationship. Additionally, by pairing a non-functional tool with a non-reward situation, participants might choose the correct tool based on its desirability and prior reward history, rather than flexibly thinking about the future [28]. Importantly, influential experimental criteria have been proposed to guide the design of behavioural tests measuring flexible planning in human and other animals [7,29]. Notably, researchers recommended the use of single trials and novel problems to separate the temporal and spatial contexts between the present future-oriented decision and the anticipated future problem. Note that for Kabadayi and Osvath's [22] study, the same task was used during training and testing, rather than a novel problem that precludes learning. Furthermore, the ravens were not given any prior reason to expect to use the selected items in a future event [28].

Therefore, the aim of our study was to design a novel experimental paradigm that addresses some of the critiques of some previous related human and non-human studies in order to test flexible planning in young children. It was important that our novel methodology could potentially be used comparatively to test non-human species, thereby addressing the critiques mentioned above. Differences in testing methodologies, such as the number and length of delays [30] can make it difficult to make comparisons between human and non-human studies. Therefore, we ensured that the apparatus and trials were designed in such a way that they could be used with humans as well as animals, such as tool-using corvids. Within this novel flexible planning task, we aimed to test delay of gratification by asking children to choose between an immediately available reward of lower quality or a tool to obtain a delayed reward of higher quality. Importantly, we also provided training trials with delays to provide experience of a predictable return of reward situation. Unlike most standard child development studies, we incorporated two delays to be in line with most non-human animal research—one delay following apparatus presentation, before the critical tool choice and one delay after the tool choice before tool use. This was different from most previous child studies that incorporated delays (e.g. 5 min to 24 h) only between the problem and selection, though no delay between selection and use [7,9]. In the testing trials, children were asked to choose between two tools for which they had equal prior positive stimulus–reward experience, only one of which was functional in a particular condition.

We tested whether 3- to 5-year-old human children could use information from past events to guide their present choice to address anticipated future problems. The aim being to validate our novel flexible planning paradigm by first testing children, where there is already extensive established flexible planning developmental research using other paradigms. We aimed to explore performance in this novel flexible planning task in relation to performance in standardized executive function and language ability tasks as part of this validation.

As Redshaw and Suddendorf [8] noted, planning for an immediate problem is less difficult than for a deferred future problem as the latter requires more executive resources. Executive function refers to a set of higher-order cognitive abilities which regulate goal-directed actions and adaptive responses in novel and complex situations [31]. Adopting factorial analytic approach, researchers have identified three key components of executive factors in adults: inhibitory control, working memory and cognitive flexibility [32]. One model, influenced by the work of Miyake *et al.* [32], along with other developmental research, indicates that in young children, executive function may be a more unitary construct comprising inhibition, working memory and cognitive shifting/flexibility—though driven primarily by inhibitory control [31,32]. Notably, inhibition and cognitive flexibility may be the most relevant cognitive functions that underlie episodic foresight, as flexible planning requires the need to focus on the simulated events, while suppressing ongoing events in the present [33]. Research on the development of executive function further suggests that in early childhood individuals vary greatly in their performance on a range of tasks tapping into different aspects of executive function, yet these measures can be loaded onto a single latent factor in early development and in the preschool year [34,35]. For the current study, we chose five age-sensitive and appropriate executive function tasks aiming to measure inhibitory control (two tasks), cognitive flexibility (shifting) (one task) and working memory (two tasks) as well as one language ability task.

Children's executive function emerges throughout preschool years and a theoretical relation with episodic foresight has been suggested [36,37]. Similarly, children's language ability has been associated with future thinking skills. A few studies empirically investigated these links and yielded mixed findings [38–40]. For example, Unal and Hohenberger [38] gave 3- to 5-year-old children an episodic memory and an episodic future thinking task, as well as three tasks to measure temporal language, executive function (inhibition) and spatial working memory. They found that after controlling for age, performance on the episodic memory and episodic future thinking task were correlated, and scores on the executive function and language task correlated with the episodic memory task, while only the language task results correlated with the episodic flexible planning task. Ford and colleagues [40] gave 4- to 6-year-old children a prospective memory task, as well as one task assessing verbal ability, five tasks assessing inhibitory control, one task assessing working memory, six tasks assessing theory of mind and one task assessing empathy. Theory of mind is defined as the ability to understand and attribute mental states to self and others and requires the change of perspectives [41]. Four out of the five inhibitory control tasks were highly correlated. They found that an aggregate score from the inhibitory control tasks, as well as an aggregate score from the theory of mind tasks, predicted the children's performance on the prospective memory task.

In another study, Hanson and colleagues [39] gave 3- to 5-year-old children a battery of five tasks measuring episodic foresight and seven tasks measuring the key aspects of executive function, namely, inhibitory control, cognitive flexibility, planning generativity and working memory. After controlling for age and language, they found that the executive function scores (individually or as a composite score) were not correlated with episodic foresight [39]. Given that previous studies have used children of different age groups, different tasks and different methods of analysis and found conflicting results, the relationship between executive function, language ability and flexible planning requires further investigation.

Thus, our aim was to test flexible planning in young children using a novel task that could be used comparatively and addressed some of the critiques of previous non-human animal paradigms. We then examined the relationship between flexible planning and executive function and language performance. As these executive function and language tasks are standardized child development tasks, we expected age to positively influence performance in these tasks, as per prior research. With our novel flexible planning task, we expected a significant age difference between 3-year-olds and older children, in line with previous child developmental research using standard flexible planning paradigms. Finally, we expected performance in the executive function, language ability and the flexible planning task to correlate positively with one another across and within each age group.

## 2. Methods

### 2.1. Participants

Participants were 87 children aged between 3 and 5 years old: 30 3-year-olds (mean: 3.82 years; range: 3.3–3.99 years), 28 4-year-olds (M: 4.53 years; R: 4.03–4.99 years) and 29 5-year-olds (M: 5.49 years; R: 5.1–5.88 years), of which 44 were male and 43 were female. Children were recruited and tested at five nurseries and primary schools in Cambridgeshire, with piloting completed at two additional schools, serving predominantly white, middle-class communities between February and March 2018. Participants were presumed to be typically developing children (an assumption supported by the teaching staff at the facilities). All children in this sample had previously been tested in a second study using a tool-use task with no flexible planning element [42]. Children were tested in temporary visual isolation from other people, or, for some of the younger children, with a staff member present who did not interact with the child. Eighty-seven children took part in the flexible planning task and the language task, 86 children took part in the dimensional change card sort (DCCS) and forwards digit task, 85 children took part in the day–night task and 84 children took part in the knock-tap and the backwards digit span tasks.

### 2.2. Flexible planning task

#### 2.2.1. Apparatus

**Figure 1.**
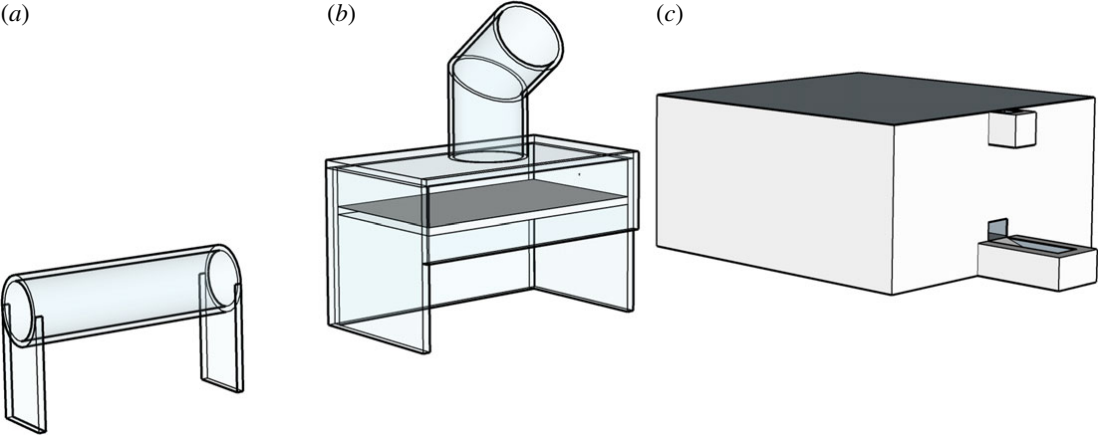
(*a*) Horizontal tube with stick tool, (*b*) drop-down with stone tool and (*c*) remote-controlled feeder apparatus with paperclip tool.

We used three different apparatuses—each apparatus had one type of functional tool, with the tools selected to be familiar to the participants (figure 1). The horizontal tube apparatus (figure 1*a*) comprises a transparent tube with two open ends. The reward rested in the centre of the tube and could be retrieved by inserting a pencil to rake or push the reward through either opening. The drop-down apparatus (figure 1*b*) comprises a transparent box with a collapsible platform and an open vertical tube. Dropping a stone of sufficient weight into the vertical tube caused the platform to collapse and release the reward. The feeder apparatus (figure 1*c*) was remotely controlled by the experimenter, and dropping a paperclip into a designated tube would release the reward. We used stickers for the most preferred (picture sticker) and least preferred (white sticker) rewards, with a medium quality sticker (yellow smiley sticker) for some parts of the pre-experience steps. Trials were timed with a stop-watch, and we used two separate locations (A and B), which were either two adjacent rooms or two areas separated by visual barriers, depending on the space available at each school.

#### 2.2.2. Procedure

Participants were tested in two sessions: in session 1, participants received several pre-experience steps and in session 2, participants received training and test trials. Session 1 lasted 8–10 min and was run in the same order for all participants. Session 2 lasted approximately 30 min and condition order was counterbalanced across participants. Eighty of the 87 test participants received the training and test session on the same day with a minimum interval of 1 h. Due to school arrangement and availability of participants, 7 children received the test session 1 or 2 days after the training session.

#### 2.2.3. Session 1 (pre-experience phase)

Step 1, tool transport: participants were asked to select a tool in location A and then use it immediately on an apparatus in location B. They were not required to make a choice as only the functional tool was presented, and they received one trial per apparatus. The apparatuses were baited with the medium quality reward.

Step 2, reward preference test: participants were asked to select the ‘best’ sticker from a choice between the most and least preferred options. The stickers were presented in front of the participants and the position (right/left) of the stickers was counterbalanced across trials. The reward preference task was conducted twice, with one trial during the pre-experience phase in session 1 and one trial at the end of testing in session 2. All children preferred the picture stickers over the white stickers.

Step 3, delay of gratification: the drop-down apparatus was baited with the most preferred reward and participants were presented with a choice between the least preferred reward and the functional tool (stone). Participants were required to select the tool to access the most preferred reward over the immediately available least preferred reward in two consecutive trials. If they failed to select the tool in these two trials they received two additional trials before proceeding to the next step.

Step 4, tool functionality: the medium reward was placed inside one of the three apparatuses and participants were asked to select the functional tool from a choice of two tools (one functional, one non-functional). Participants received two trials per apparatus, with three additional trials run if they failed, i.e. selected the incorrect tool in any of the previous trials, starting with the apparatus that they failed on. The presentation order of apparatuses and the location of tools were counterbalanced across participants and trials.

**Table 1.** Training and test conditions for flexible planning task.

condition	1	2	3	4
trial type	training	training	test	test
apparatus	drop-down	drop-down	horizontal tube	remote-controlled feeder
reward type inside apparatus	most preferred	least preferred	most preferred	most preferred
items presented	stone versus least preferred reward	stone versus most preferred reward	pencil versus paperclip	pencil versus paperclip
correct choice	stone	most preferred reward	pencil	paperclip

#### 2.2.4. Session 2 (training and test trials)

**Figure 2.**
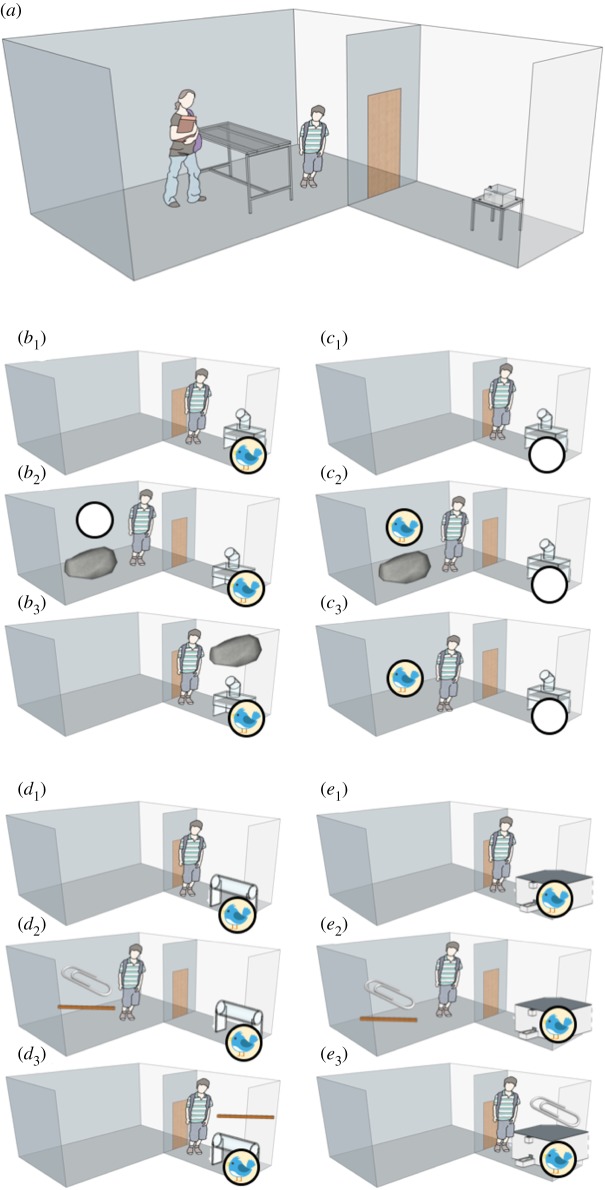
Procedure of training and test trials for flexible planning task. (*a*) General set-up: experimenter and participant in room 1, apparatus out-of-sight in room 2. Step 1: in room 2, participant has visual access for 10 s to apparatus containing reward (training: (*b*) most preferred reward in drop-down apparatus, (*c*) least preferred in drop-down apparatus; testing: (*d*) most preferred in horizontal tube apparatus, (*e*) most preferred in feeder apparatus). Step 2: after a delay, participant makes choice of tool or tool/sticker in room 1 (training: (*b*) choice = stone versus least preferred reward, (*c*) stone versus most preferred reward; testing: (*d,e*) stick versus paperclip. Step 3: after a delay, participant has access to baited apparatus in room 2—can bring their chosen item to use with apparatus (tool/sticker).

Participants received two training conditions and two test conditions, with one trial per condition (table 1). The training conditions provided participants with experience of a predictable return of reward situation in a future event. The test trials investigated whether participants could select the correct tool to solve an anticipated future problem. In each condition, participants were first shown the baited apparatus in location *a* (figure 2, steps *b*_1_, *c*_1_, *d*_1_ and *e*_1_). Participants then waited in location *b* for a set delay time, after which they could select one of two items (training trials: tool or reward, test trials: choice between a functional and a non-functional tool) to use later (figure 2, steps *b*_2_, *c*_2_, *d*_2_ and *e*_2_). After a second delay, they returned to location *a* and could use this item to try to access the reward from the apparatus (figure 2, steps *b*_3_, *c*_3_, *d*_3_ and *e*_3_). In the training conditions, the reward inside the apparatus was either high quality or low quality, and the children made a choice between the corresponding reward and the functional tool (figure 2, conditions *b* and *c*). In the training conditions, though not in the test conditions, we incorporated a short verbal cue prior to the item choice, indicating that we would be returning to location *a* later.

In the test conditions, the most preferred reward was inside the horizontal tube or remote-controlled feeder apparatus (figure 2, conditions *d* and *e*). Participants had no prior experience of a delay between tool choice and use for these two test apparatuses, though they were otherwise equally familiar with these apparatuses and their respective tools from session 1 pre-experience (figure 2). They therefore had comparable experience of obtaining a reward using each tool with each apparatus. In the test conditions, the same two tools were presented in each trial (pencil and paperclip); therefore, one tool was correct in one condition and the other tool was correct in the other condition. In all conditions, we incorporated two delays of different length between steps 1–2 and steps 2–3 (2 and 5 min; figure 2). Participants were split into two subgroups with half of the participants per age in each one: subgroup A received a 2 then 5 min delay; subgroup B received a 5 then 2 min delay, with the delay order consistent across conditions within participant. The order of conditions within training and testing conditions was counterbalanced across participants. Testing was conducted by R.M., A.F. and N.D., following a script and prior training by R.M. The electronic supplementary material, Movie shows example clips of testing.

### 2.3. Executive function and language tasks

We conducted several executive function tasks designed to test inhibition, cognitive flexibility and working memory, and a language ability measure (table 2). Specifically, these were: (1) inhibition = knock-tap, day–night, (2) cognitive flexibility = DCCS, (3) working memory = forwards and backwards digit span, and (4) receptive language = British picture vocabulary scale. We outline the task administration protocols further in the electronic supplementary material. We ran these tasks within the delays in the flexible planning task, in a set order within subgroups A and B.

**Table 2.** Task descriptions for executive function and language ability.

task		Description
inhibition	knock-tap [43]	Participants were asked to perform the opposite hand movement from the experimenter, so if the experimenter knocked on the table, the subject should tap the table with their flat palm and vice versa. The number of correct responses (out of 15 trials) was recorded.
	day–night [44]	Participants were first asked to identify the day (picture of a sun) and night cards (picture of a moon), then were asked to say ‘day’ when shown the night card and ‘night’ when shown the day card. Cards were shown individually in random order, and the number of correct responses (out of 16 trials) was recorded.
cognitive flexibility	DCCS [45]	Participants were asked to sort 12 cards in each set based on a rule for example colour of the pictures shown on the cards. After six cards, the rule was then switched so that they were required to sort by a different rule, for example shape. Half the participants started with colour and switched to shape, and half started with shape and switched to colour. The number of correct responses in the post-switch phase was scored.
working memory	forwards digit span	Participants were asked to repeat a series of single digits in order after they were read out by the experimenter. For example, 6–9, 5–8–2, 5–2–8–3, 1–3–6–2–9. The highest number of digits recalled was recorded.
	backwards digit span [46]	Participants were asked to repeat a series of single digits in reverse order after they were read out by the experimenter. For example, 3-5, 4-9-5, 1-9-6-2, 7-3-5-1-9. The highest number of digits recalled was recorded.
receptive language	British picture vocabulary scale—3rd edition [47]	Participants were asked to identify pictures corresponding to spoken words from the experimenter. Each participant received a number of vocabulary sets depending on their language ability. Raw and standardized scores were calculated using the manual.

### 2.4. Data analysis

We recorded the choice per trial for each participant as ‘correct’ or ‘incorrect’. All test sessions were coded live as well as being video-recorded unless parental consent requested otherwise. Ten percent of trials were coded from video and compared with the live coding, finding 100% agreement with the data.

We conducted generalized linear mixed models (GLMM: [48]) using R version 3.6.1 [49] to assess which factors influenced success rate in the flexible planning task in children. Success was a binary variable indicating whether the participant correctly solved the trial (1) or not (0) and was entered as a dependent variable in the models. We included the random effect of participant ID, fixed effects of age in years (continuous: ages 3–5 in individual years), condition (1–2 training, 3–4 for testing), gender (male/female), delay order (short-long delay/ long-short delay) and training performance (solved—1, not solved—0, for model 1 only). We ran two separate models: (1) test trials and (2) training trials. We used likelihood ratio tests to compare the full model (all predictor variables, random effects and control variables) firstly with a null model, and then with reduced models to test each of the effects of interest [50]. The null model consisted of random effects, control variables and no predictor variables. The reduced model comprised all effects present in the full model, except the effect of interest [51]. We then ran further *post hoc* comparisons using exact Mann–Whitney *U*-tests and Wilcoxon signed-rank tests.

Using R [49], we also assessed whether age had an effect on each executive function task and language ability using Kruskal–Wallis tests from the *pastecs* package [52]. We then ran *post hoc* tests for the Kruskal–Wallis tests to examine how the age groups differed using the *pgirmess* package [53]. We tested whether individuals' success on the flexible planning test correlated with their score in each of the executive function and language ability tasks, using Spearman correlations, with a Bonferroni correction, in the *psych* package [54].

## 3. Results

### 3.1. Children's performance in the flexible planning task

In the test trials, the full model differed significantly from the null model (*χ*^2^ = 11.46, d.f. = 5, *p* = 0.04). We found a significant main effect of age (*χ*^2^ = 4.85, d.f. = 1, *p* = 0.027) on success rate in the two test trials, with success increasing with age (electronic supplementary material, table S1). Specifically, 3-year-olds performed significantly more poorly than 4- and 5-year-olds (Mann–Whitney *U*-test: 3 versus 4 years: *U* = 1344, *z* = −2.24, *p* = 0.032: 3 versus 5 years: *U* = 1407, *z* = −2.16, *p* = 0.035), with no significant difference in success rate between 4- and 5-year-olds (*U* = 1610, *z* = −0.105, *p* > 0.999). In the test trials, 3-year-old children did not significantly select correctly above chance, while children aged 4 and 5 years did significantly select correctly above chance (table 3, electronic supplementary material, table S2).

**Table 3.** Mean score ± s.d. in all tests for each age group.

age group	flexible planning test trial out of 2	knock-tap out of 15	DCCS (post-switch) out of 6	day–night out of 16	numbers forwards digits achieved	numbers backwards digits achieved	BPVS-3 verbal raw score
3	1.10 ± 0.66	10.79 ± 5.66	3.62 ± 1.76	10.95 ± 4.90	4.00 ± 0.87	1.28 ± 0.59	56.96 ± 14.58
4	1.50 ± 0.51	13.07 ± 3.54	4.50 ± 1.77	12.61 ± 4.16	4.39 ± 0.69	1.73 ± 0.83	62.96 ± 13.07
5	1.48 ± 0.79	13.45 ± 2.65	5.03 ± 1.12	13.82 ± 2.55	4.38 ± 0.86	2.41 ± 0.95	77.14 ± 14.38

In the training trials, the full model differed significantly from the null model (*χ*^2^ = 36.59, d.f. = 2, *p* ≤ 0.001). We found a significant main effect of condition (*χ*^2^ = 6.44, d.f. = 1, *p* = 0.011) on success rate (correct versus incorrect choice) in the two training trials (electronic supplementary material, table S3). In condition 1, the least preferred reward was inside the apparatus, and the choice was between the most preferred reward and the functional tool with the correct choice being the immediately available reward. In condition 2, the most preferred reward was inside the apparatus, and the choice was between the least preferred reward and functional tool, with the correct choice being the immediately available tool. Across all ages, children showed significantly higher success in condition 2 than 1 (Wilcoxon signed-rank test: *Z* = −2.33, *p* = 0.029). In the training trials, children aged 3–5 years significantly selected correctly above chance (electronic supplementary material, table S2).

### 3.2. Children's performance in executive function and language ability tasks

Age had a significant effect on all executive function scores and language ability score, except for the knock-tap task (table 3, electronic supplementary material, table S4). Five-year-olds outperformed 3-year-olds except on the forwards digit task (all *p* < 0.05). Additionally, 5-year-olds scored significantly higher on the backwards digit span task and language ability than 4-year-olds (all *p* < 0.05). Across all ages and within the executive function tasks only, performance in the knock-tap task correlated with numbers forwards digits task, DCCS correlated with day–night and numbers backwards digits, day–night correlated with numbers forwards digit, and numbers forwards digits correlated with numbers backwards digit task (table 4). Language performance correlated with all executive function tasks except the knock-tap task (table 4).

**Table 4.** Spearman's rank-order correlation between flexible planning, executive function tasks and language ability across all ages. We include the correlation coefficient, its 95% confidence interval, the sample size and the *p*-values. Sample sizes (*n*) vary as not all children took part in all of the tasks. Significant *p*-values are highlighted in bold.

task	knock-tap	DCCS (post-switch)	day–night	numbers forwards digits	numbers backwards digits	BPVS-3 verbal raw score
flexible planning	0.11 (CI: −0.11; 0.31) (*n* = 84, *p* = 1.00)	0.13 (CI: −0.08; 0.33) (*n* = 86, *p* = 1.00)	0.16 (CI: −0.07; 0.35) (*n* = 85, *p* = 1.00)	−0.04 (CI: −0.51; 0.17) (*n* = 86, *p* = 1.00)	0.11 (CI: −0.10; 0.32) (*n* = 84, *p* = 1.00)	0.20 (CI: −0.01; 0.40) (*n* = 87, *p* = 1.00)
knock-tap	—	0.20 (CI: −0.01; 0.40) (*n* = 84, *p* = 1.00)	0.33 (CI: 0.12; 0.51) (*n* = 83, *p* = 0.056)	**0.36 (CI: 0.16; 0.53) (*n* = 84, *p* = 0.017)**	0.28 (CI: 0.07; 0.47) (*n* = 82, *p* = 0.218)	0.29 (CI: 0.08; 0.47) (*n* = 84, *p* = 0.173)
DCCS (post-switch)		—	**0.33 (CI: 0.13; 0.51) (*n* = 85, *p* = 0.042)**	0.27 (CI: 0.06; 0.46) (*n* = 86, *p* = 0.239)	**0.34 (CI: 0.13; 0.52) (*n* = 84, *p* = 0.034)**	**0.32 (CI: 0.12; 0.50) (*n* = 86, *p* = 0.048)**
day–night			—	**0.34 (CI: 0.14; 0.52) (*n* = 85, *p* = 0.029)**	0.32 (CI: 0.12; 0.50) (*n =* 83, *p* = 0.060)	**0.42 (CI: 0.23; 0.58) (*n* = 85, *p* = 0.001)**
numbers forwards digits				—	**0.41 (CI: 0.21; 0.57) (*n* = 84, *p* = 0.002)**	**0.35 (CI: 0.15; 0.52) (*n* = 86, *p* = 0.022)**
numbers backwards digits					—	**0.61 (CI: 0.45; 0.73) (*n* = 84, *p*<0.0001)**

### 3.3. Relationship between executive function tasks, language ability and flexible planning task performance

Across all ages, we found that performance in the flexible planning task was not correlated with any of the executive function or language ability tasks (table 4). Within age groups, performance in the flexible planning task did not correlate with any executive function or language ability measures for any of the age groups (electronic supplementary material, table S5).

## 4. Discussion

In the current study, we used a novel behavioural paradigm featuring different types of tools, rewards and apparatuses to examine young children's ability to use past information to solve a future problem. We then related performance in the flexible planning task with several executive function and language ability tasks. Specifically, in the flexible planning task, the children were presented with apparatuses containing a reward in location 1. The findings revealed significant age-related differences in flexible planning, executive function and language performance. In the flexible planning task, children aged 4 and 5 successfully linked past events to deferred future episodes and made future-directed decision in this tool-use context, while 3-year-olds were unable to do so. We found similar age-related differences in children's performance in the executive function and language tasks. While performance in many of these tasks correlated with each other, there was no correlation between performance in any executive function and the flexible planning task. Similarly, language ability was also not correlated with performance in the flexible planning task.

Our results were consistent with most previous research which reported similar developmental trajectory of flexible planning skills using standard child development paradigms [7,8,13,55]. There was no relationship between delay length and performance in the flexible planning task. Notably, unlike other paradigms, we incorporated two delays. In our study, half of the participants waited 2 min and the other half waited 5 min in the first delay. This was different to most previous child studies that incorporated delays (e.g. 5 min to 24 h) between the problem and selection, though no delay between selection and use [7,9]. In comparison, non-human studies typically include a delay after the selection phase, though not before [30]. As we aimed for this paradigm to be useful for comparative work, we included delays both prior and following the critical selection stage. Atance and Sommerville [9] also explored the effect of a delay between selection and use in children and found no effect on performance in 4-year-olds, though they did not test 3-year-olds. In our study, we found that a delay as short as 2 min between problem and selection, or between selection and use, could pose adequate difficulty for 3-year-olds. This finding is in line with most previous child development work where 3-year-olds were only able to solve problems that were of immediate concern, reflecting difficulty linking past events to deferred future episodes [7,8].

Most research indicates that 4-year-olds pass flexible planning tests, including the present study. However, Dickerson and colleagues found that, when presenting participants with a choice of two high-value objects after seeing a specific problem, 4-year-olds failed to pass the test [12]. In our study, the choice in the test trials was similarly between two objects of equal value, only one of which was functional in a particular condition, and we found that 4-year-olds did pass the tests, though notably with a short delay. The next step would be to further manipulate delay length and include other possible options within the choice that have similar value, like tools that are rewarded in other contexts or immediately available rewards.

We ran this study with the aim of testing flexible planning in young children using a novel paradigm that addressed some of the key critiques of non-human animal studies, and relating flexible planning performance to performance in executive function and language tasks. The current study therefore has several methodological advantages with this flexible planning task. First of all, we used a single trial design to avoid repeated reward-stimulus relationships. Participants received one test trial for each apparatus to assess their tool choice specific to that condition. Secondly, children had equal experience with both tools being functional as the paperclip and pencil had been equally associated with reward in the pre-experience phase. Thus, there was no difference in desirability of tools and the correct choice in test trials depended on the apparatus presented. Therefore, the task effectively tapped into children's ability of linking past information (type of apparatuses) to future-directed decisions (type of tools). We used training trials to establish the experience of predictable return of future reward, which allowed us to attribute children's performance on tool selection to future-directed cognitive processes. As recommended by previous research, we used a single trial for each condition, and novel problems in the test sessions compared to the training sessions which precludes learning [7,29]. Additionally, unlike most developmental research, we did not use verbal cues during testing making the current paradigm potentially suitable for comparative studies. Limitations of this study include the cross-sectional design, a sample drawn only from the UK with no monitoring or attempt to diversify socio-economic status, and seven out of the 87 children were tested on a different day for the training sessions. Future studies may test children multiple times at different ages to measure the development of inhibition and executive function within each participant. Finally, as the children performed similarly in our flexible planning task as in previous developmental studies [14], this may indicate validity of this novel paradigm for testing flexible planning in other species, specifically corvids, such as tool-using New Caledonian crows, or primates.

We also examined the relationship between children's flexible planning, executive function and language ability. We adopted a battery of standardized executive function measures and found, as expected by previous research [56], significant effects of age on performance, except for in the knock-tap task. Across all ages, several executive function measures were significantly correlated with one another and with the language ability measure. Previous research found correlations between flexible planning tasks and language ability, and these tasks had a high reliance on language in their flexible planning tasks [38,40]. Our data showed correlations in performance between the children's language ability and all executive function tasks apart from the knock-tap task, which was the only task that did not require language. Our flexible planning task was primarily non-verbal in the test trials and flexible planning performance did not correlate with language ability in any age group. This indicates that this task may be used with non-verbal species as well, making it easily comparable.

We found no significant relationship between flexible planning performance and executive function measures across all ages, which may be due to the nature of our flexible planning task, as other studies also found no inter-correlations between flexible planning and executive function task performance using a different test paradigm [39]. As suggested in Hanson and colleagues' study [39], it is possible that the association between executive function and episodic foresight may only exist in flexible planning tasks that feature conflicts between present and future states. However, Ford and colleagues [40] found a correlation between inhibitory control and flexible planning, using two tasks that are similar to the knock-tap task: the hand shape task and the tapping task.

One model indicates that executive function may be a unitary construct with partially dissociable components [32,34,35,57,58] including inhibition, working memory and cognitive shifting/flexibility—though driven primarily by inhibitory control [31,32]. In previous studies, different tasks have been used to measure these three components, which may explain the variation in results. Some studies have also used composite scores of executive function tasks to examine the correlation between executive function and flexible planning (e.g. [39]). This may contribute to the lack of correlation between executive function and flexible planning that has been found in some previous research, as despite correlating with one another, it is possible that only specific aspects of executive function, like inhibition, may correlate with performance in some flexible planning tasks. In training trials for the flexible planning task, children of all ages were able to inhibit the selection of an immediately available low-value reward for a tool in order to obtain a higher value reward inside the apparatus. They could also correctly select the high-value reward when it was immediately available. However, they did perform better when the high-value reward was inside the apparatus and the correct choice was the tool, indicating that children of all ages showed a general interest in tool use. Therefore, it is likely that tool use itself was rewarding for the participants, indicating that step 3 in session 1 and the training steps in session 2 may not have measured delay of gratification, but rather the participants' ability to make a correct choice when presented with two options.

In conclusion, we used a novel experimental paradigm to test flexible planning ability in children, which could be run comparatively with other non-human species, while addressing critiques of some previous related animal studies. We then related flexible planning performance to performance in several standardized executive function and language tasks. The flexible planning task appeared to successfully tap into pre-schoolers’ ability of mentally linking past events to deferred future events and to make future-directed decisions. We detected significant age-related differences in performance in children's flexible planning ability and the developmental trajectory echoed those from standard developmental studies. These findings indicate that this may be a valid test for exploring flexible planning in the context of tool use in humans and other species.

## Supplementary Material

Supplementary Material

Reviewer comments

## Supplementary Material

Supplementary Video

## Data accessibility

The full dataset is available on Figshare doi:10.6084/m9.figshare.7227470.
