# Supplementary material for: A novel test of flexible planning in relation to executive function and language in young children
Source: R Soc Open Sci. 2020 Apr 15;7(4):192015. doi: 10.1098/rsos.192015 (PMC7211888; doi:10.1098/rsos.192015)
Supplement: Supplementary Material [file rsos192015supp1.docx]

**SUPPLEMENTARY INFORMATION**

**A novel test of flexible planning in relation to executive function and language in young children**

Rachael Miller^1^, Anna Frohnwieser^1^, Ning Ding^1^, Camille A. Troisi^1,4^, Martina Schiestl^2^, Romana Gruber^2^, Alex H. Taylor^2^, Sarah A. Jelbert^1,5^, Markus Boeckle^1,3^, Nicola S. Clayton^1^

^1^ Department of Psychology, Cambridge University, Cambridge, UK

^2^ School of Psychology, Auckland University, Auckland, New Zealand

^3^ Department of Psychotherapy, Bertha von Suttner University, St. Pölten, Austria

^4^ School of Biological, Earth and Environmental Sciences, University College Cork, Cork, Ireland

^5^ School of Psychological Science, University of Bristol, Bristol, UK

Supplementary Table 1. Generalized linear mixed models on factors affecting the number of correct **test** trials in the flexible planning task. N = 87. Significant p-values in bold.

| **Fixed term** | **Estimate** | **z-value** | **p-value** |
| --- | --- | --- | --- |
| **Age in years** | **0.489** | **2.176** | **0.030** |
| Condition | -0.707 | -1.998 | 0.051 |
| Gender | -0.473 | -1.308 | 0.191 |
| Delay order | -0.500 | -1.407 | 0.160 |
| Training | 0.140 | 0.337 | 0.736 |

Supplementary Table 2. Correct choices (%) in each condition for each age (3-5 years) in the flexible planning task. P-values calculated from exact two-tailed binomial tests. Significant p-values are highlighted in bold.

| **Age in years** | **Training** | | **Testing** | |
| --- | --- | --- | --- | --- |
|  | % | p-value | % | p-value |
| 3 | 68 | **0.006** | 55 | 0.519 |
| 4 | 80 | **<0.001** | 75 | **<0.001** |
| 5 | 79 | **<0.001** | 75 | **<0.001** |

Supplementary Table 3. Generalized linear mixed models on factors affecting the number of correct **training** trials in the flexible planning task. N = 87. Significant p-values in bold.

| **Fixed term** | **Estimate** | **z-value** | **p-value** |
| --- | --- | --- | --- |
| Age in years | 0.305 | 1.395 | 0.163 |
| **Condition** | **0.906** | **2.441** | **0.015** |
| Gender | 0.607 | 1.674 | 0.094 |
| Delay order | -0.348 | -0.975 | 0.329 |

Supplementary Table 4**.** Kruskal-Wallis tests on the age effect on executive function tasks and language. Significant p-values in bold.

| Task | H | df | p-value |
| --- | --- | --- | --- |
| Knock-Tap | 2.183 | 2 | 0.336 |
| DCCS | 7.592 | 2 | **0.022** |
| Day-Night | 7.140 | 2 | **0.028** |
| Forward Digit Span | 6.292 | 2 | **0.043** |
| Backward Digit Span | 24.944 | 2 | **< 0.001** |
| BPVS-3 | 26.441 | 2 | **< 0.001** |

Supplementary Table 5: Spearman’s rank-order correlation between success on the flexible planning task with performance on each of the executive function and language ability tasks, for each age group separately. We include the correlation coefficient, its 95% confidence internal, the sample size and the p-values. Sample sizes (n) vary as not all children took part in all of the tasks. Significant p-values are highlighted in bold.

| Age | Knock-Tap | DCCS | Day-Night | Forward Digit Span | Backward Digit Span | BPVS-3 |
| --- | --- | --- | --- | --- | --- | --- |
| 3 | 0.10 (CI: -0.28; 0.46) (n=28, p=1.00) | 0.02 (CI: -0.35; 0.38) (n=29, p=1.00) | -0.02 (CI: -0.37; 0.35) (n=29, p=1.00) | 0.001 (CI: -0.365; 0.368) (n=29, p=1.00) | 0.18 (CI: -0.20; 0.51) (n=29, p=1.00) | 0.09 (CI: -0.28; 0.43) (n=30, p=1.00) |
| 4 | 0.18 (CI: -0.22; 0.52) (n=27, p=1.00) | 0.06 (CI: -0.32; 0.43) (n=28, p=1.00) | 0.11 (CI: -0.27; 0.47) (n=28, p=1.00) | -0.14 (CI: -0.49; 0.24) (n=28, p=1.00) | -0.14 (CI: -0.50; 0.26) (n=26, p=1.00) | 0.19 (CI: -0.19; 0.53) (n=28, p=1.00) |
| 5 | 0.05 (CI: -0.32; 0.41) (n=29, p=1.00) | 0.15 (CI: -0.23; 0.49) (n=29, p=1.00) | 0.13 (CI: -0.26; 0.48) (n=28, p=1.00) | -0.20 (CI: -0.53; 0.18) (n=29, p=1.00) | -0.14 (CI: -0.48; 0.24) (n=29, p=1.00) | 0.03 (CI: -0.34; 0.39) (n=29, p=1.00) |

**Executive function and language test: administration protocols**

Inhibition tasks: *Knock-tap* (1)

This motor task tapped into the inhibition of immediate impulses evoked by visual stimuli which conflict with task instructions. Participants were instructed to perform the opposite hand movement from the experimenter. Specifically, they were asked to knock on the table when the experimenter taps on the table with their flat palm, and vice versa. There were two practice trials with feedback to ensure that participants understand the instructions and if they failed both practice trials no test trials would be administered. There were no rule reminders during test trials and the number of correct responses (out of 15) was recorded.

*Day-night* (2)

This ‘stroop-like’ inhibition task measured participants’ ability to act according to remembered instructions and concurrently inhibit a pre-potent response tendency. The task began with the experimenter engaging participants in a conversation about cards showing the sun and the moon. Then participants were asked to say ‘night’ for the sun card and ‘day’ for the moon card. Two practice trials were presented first to make sure that participants understand the rules and failure in both practice trials would result in no test trials being administered. Cards were shown individually in a fixed, pseudo-random order. No feedback was provided during test trials and the number of correct responses (out of 16 trials) was recorded.

Cognitive flexibility: *Dimensional Change Card Sort (DCCS)* (3)

This task used bivalent cards (colour and shape) and required participants to switch and sort cards with different rules. The experimenter first presented two target cards and asked participants to identity the colour and shape of the objects (e.g. red boat and blue rabbit). Then participants were told that they were going to sort cards by the colour/shape rule with two demonstration trials. They were given six trials in the pre-switch stage. Next, in the post switch trials, experimenter explained that sorting rule has changed and participants were given six cards to sort with a different rule. Half of the participants started with colour and switched to shape, and half started with shape and switched to colour. The number of correct responses in the post-switch phase were scored.

Working memory: *Forward Digit Span* (4)

This test was conducted as an assessment of short-term memory and as a warm-up for the following backward digit span task. Participants were asked to repeat a series of single digit in the exact same order after they were read out loud by the experimenter. For example, 6-9, 5-8-2, 5-2-8-3, 1-3-6-2-9. The highest number of digits remembered and recalled was recorded.

*Backward Digit Span* (4)

Participants were presented with a random string of single digits and were instructed to repeat the string of digits in reverse order. The strings began with two digits and a correct response led to the next string being one digit longer, for example, 3-5, 4-9-5, 1-9-6-2, 7-3-5-1-9. Participants received two practice trials with feedback prior to test trials to make sure they understand the rules. The test stopped when participants errored on two consecutive trials and the highest level of success (number of digits recalled) were recorded.

Receptive language: *British Picture Vocabulary Scale (BPVS 3rd edition)* (5)

This test measured the breadth of participants’ vocabulary knowledge. In each trial, participants were asked to select one out of four pictures that best corresponded the meaning of a word read out loud by the experimenter. Each participant received a number of vocabulary sets depending on their language ability and the test stopped when participants errored on eight out of the 12 trials within a set. The test was administered and raw and standardised scores were calculated based on the guidelines in the BPVS manual.

**References**

1. Luria A. Higher cortical functions in man. New York: Basic Books; 1966.

2. Gerstadt CL, Hong YJ, Diamond A. The relationship between cognition and action: performance of children 3.5-7 years old on a stroop-like day-night test. Cognition. 1994;53:129-53.

3. Zelazo PD. The Dimensional Change Card Sort (DCCS): a method of assessing executive function in children. Nat Protoc. 2006;1(1):297-301. PubMed PMID: 17406248. Epub 2007/04/05.

4. Davis HL, Pratt C. The development of children's theory of mind: the working memory explanation. Australian Journal of Psychology. 1996;47(1):25-31.

5. Dunn LM, Dunn DM. The British picture vocabulary scale: GL Assessment Limited; 2009.
