# Supplementary material for: A novel test of flexible planning in relation to executive function and language in young children
Source: R Soc Open Sci. 2020 Apr 15;7(4):192015. doi: 10.1098/rsos.192015 (PMC7211888; doi:10.1098/rsos.192015)
Supplement: Reviewer comments [file rsos192015_review_history.pdf]

THE ROYAL SOCIETY
PUBLISHING

ROYAL SOCIETY
OPEN SCIENCE

# A novel test of flexible planning in relation to executive function and language in young children

Rachael Miller, Anna Frohnwieser, Ning Ding, Camille A. Troisi, Martina Schiestl, Romana Gruber, Alex H. Taylor, Sarah A. Jelbert, Markus Boeckle and Nicola S. Clayton

**Article citation details**
*R. Soc. open sci.* **7**: 192015.
http://dx.doi.org/10.1098/rsos.192015

**Review timeline**

| | |
|---|---|
| Original submission: | 15 May 2019 |
| 1st revised submission: | 18 November 2019 |
| 2nd revised submission: | 27 February 2020 |
| Final acceptance: | 10 March 2020 |

Note: Reports are unedited and appear as submitted by the referee. The review history appears in chronological order.

# Review History

## RSOS-190894.R0 (Original submission)

### Review form: Reviewer 1

**Is the manuscript scientifically sound in its present form?**
No

**Are the interpretations and conclusions justified by the results?**
No

**Is the language acceptable?**
Yes

**Is it clear how to access all supporting data?**
Yes

**Do you have any ethical concerns with this paper?**
No

Reports © 2020 The Reviewers; Decision Letters © 2020 The Reviewers and Editors; Responses © 2020 The Reviewers, Editors and Authors. Published by the Royal Society under the terms of the Creative Commons Attribution License http://creativecommons.org/licenses/by/4.0/, which permits unrestricted use, provided the original author and source are credited

**Have you any concerns about statistical analyses in this paper?**
Yes

**Recommendation?**
Major revision is needed (please make suggestions in comments)

**Comments to the Author(s)**
Summary
This is an interesting study, using innovative and age-appropriate tasks, which will be of particular value to researchers interested in using common measures in comparative research with human and non-human animals. Given the current emphasis in the paper on the association between flexible planning and EF, I recommend that that the statistical tests used are corrected for multiple comparisons and the discussion reframed accordingly.

Major comments
1. SM Table 5 (and all related discussion and conclusions): Correction should be made for multiple comparisons – and the discussion adjusted accordingly. Knock-tap does not correlate with flexible planning performance within any of the age bands; this should be referred to in discussion.

2. Please present plots of the key correlations referred to in the discussion (i.e. Knock-tap with flexible planning). Do ceiling effects in the planning task influence the association?

3. Lines 196-200: In the explanation of the Session 1 (pre-experience phase) it is not clear to me how the most- and least-preferred stickers are defined. If the child selects the (researcher-defined) 'low quality' white sticker, does that make it the most-preferred option for that child? If not, why not? If the child does not show a preference for the 'high quality' sticker is that accounted for in step 3?

4. The variable labels in the supporting data do not make it clear which is the variable used for 'flexible planning' (in Supplementary table 5); please clarify. Further, I have not been able to reproduce the correlation matrix presented in Supplementary table 5 and sample sizes vary from that presented (e.g. in the supporting data provided, n=86 participants contributed knock-tap data and forward digit span data, but only n=84 are presented in Supplementary table 5.

Minor comments

5. Line 147. Please provide a justification (and references) for your characterisation of the different tasks as distinctly capturing inhibitory control, cognitive flexibility and working memory

6. Line 189. Please present the number of children with an inter-session interval greater than two hours. It seems likely that the inter-session interval might influence performance – please elaborate on this in the supplementary materials.

7. Line 201: I am not convinced that step 3 of the session 1 procedure can truly be characterised as delay of gratification if, as is indicated in the discussion on p12 line 365, children find tool use gratifying in itself. Please justify this characterisation or reframe step 3. Related to this, it might be informative for future studies (not expected here) to have a condition in which the kit is not baited; do participants still drop the stone in just because it is fun?

8. I would find it useful to have the correlations currently presented in Supplementary Table 5 within the main manuscript. The table would also benefit from clearer formatting/layout.

9. The correlation between flexible planning and language ability has not been corrected for multiple tests) and may be a false positive; this should be considered in the discussion

10. Lines 354-356: I think that the statement that understanding of 'yesterday' and 'tomorrow' is not yet established in 3 year olds is an overly broad assertion if based only on the Harner study cited (which features just 30 3-year-olds, with 1 paradigm, and does not report the proportions of 3-year-olds who do and do not understand these concepts). Please provide further support for this statement (or omit it from the discussion).

11. Lines 360-361: The statement that 'children of all ages were able to inhibit the selection of an immediately available low-value reward for a tool in order to obtain a higher value reward inside the apparatus' needs some unpacking/reframing. There is an implication here that there was an objectively better sticker; returning to my previous comment re line 196-200 what if the child preferred white stickers? Secondly, an inhibitory demand would be expect to lead to a performance cost yet participants performed better when the high-value reward was inside the apparatus. Thirdly, in my view the lack of association between the day/night task (which is described here as an inhibitory control measure) and flexible planning performance indicates that the demands of the task are not primarily inhibitory.

12. Lines 383-384: This sentence is self-contradictory; EF is either unitary or has 3 components. But either way I am not convinced that the literature currently supports a strong claim in either direction. The Miyake et al. (2000) paper was followed by a study by Miyake and Friedman (2012) demonstrating that, in adolescents and young adults, two dissociable EF components can be identified, as well as a 'common EF' component that is shared across tasks (and it should be noted, that this model is specific to the tasks used; the authors acknowledge that different tasks might yield different conclusions). Amongst pre-schoolers it has been argued that a single latent EF construct best describes pre-schoolers' performance on EF batteries (Hughes, Ensor, Wilson, & Graham, 2009; Senn, Espy, & Kaufmann, 2004; Wiebe, Espy, & Charak, 2008; Wiebe et al., 2011; Willoughby, Wirth, Blair & Family Life Project Investigators, 2012), and that EF factors can be dissociated (Bernier, Carlson, Deschênes, & Matte-Gagné, 2012; Garon, Smith, & Bryson, 2014; Mulder, Hoofs, Verhagen, van der Veen, & Leseman, 2014; Skogan et al., 2015).

13. Line 393: It should be clarified that in this study one 1 inhibition task correlated with flexible planning performance

# Review form: Reviewer 2

**Is the manuscript scientifically sound in its present form?**
Yes

**Are the interpretations and conclusions justified by the results?**
No

**Is the language acceptable?**
Yes

**Is it clear how to access all supporting data?**
Yes

**Do you have any ethical concerns with this paper?**
No

**Have you any concerns about statistical analyses in this paper?**
No

**Recommendation?**
Major revision is needed (please make suggestions in comments)

**Comments to the Author(s)**
Comments to the Author

This is my first review of the manuscript entitled "Executive function, language and flexible planning in young children."
This cross-sectional research builds on a new experimental tool-use paradigm designed to assess flexible planning in 3 to 5 year olds. The task is designed to address particular flaws that have been observed in earlier studies on this topic. Particularly, single trials were used to avoid repeated exposure to stimulus-reward relationships, training trials provided the participants with experience of a predictable return of reward, children were not verbally cued during the task, and delays were included before and after tool choice. Relations between flexible planning performance in this paradigm and receptive language as well as executive functioning were also examined. See comments to authors below.

Title: My understanding is that the flexible planning is the main part of this study and therefore I think it would be more descriptive with a title like, Flexible planning in 3-5 year old children and relations

Introduction
The introduction is relatively well-written, but would gain from a re-write/structure in relation to the order of the aims of this study, which appears to be several and somewhat unclear and unstructured. I would recommend the authors to think through what the main, second and third aim etc of this study really are and structure the introduction in the same order. To my understanding the following aspects are included in aims: to design a task that can be comparable with non-human studies, to design a task that addresses flaws in earlier paradigms on the same topic ( see paragraph above), to examine the role of delay of gratification in flexible planning, to examine relations with language and several executive functions. Please order these according to their significance and re-structure the introduction correspondingly.
Surprisingly, there are no predictions/ hypotheses formulated regarding the research questions asked. Please add predictions in relation to each research questions under the aims section.
A minor ( yet important) point in the introduction ( and discussion) is that the authors describe executive functions as a unitary function. As is empirically established by Miyake and others ( who the authors also refer to) EF is a complex and very broad higher order cognitive construct that show both unity and diversity and should be referred to accordingly. Please change.

Methods

Subjects. The sample should be described more thoroughly. What was the inclusion/exclusion criteria? Were they all typically developing? Born after week 36? Etc. Measures of SES should be included ( for example; income and parents ‘education level).
Apparatus. Although the supplementary movie and figures were helpful in understanding the apparatus and procedure. Indeed, the apparatus and procedure used is quite complex and the authors need to make sure that it is clearly described in the manuscript to ease the understanding of the readers. For example, make sure to describe the three different apparatuses used in the same or that they are illustrated in Fig 1. Or if described in the same order as they were used, please change order of picture accordingly. I had a hard time understanding the apparatus and the different steps in the procedure and had to read these sections many times. Please think through how this section can be described even more clearly, so the reader can understand it without having to watch the movie.

Procedure
On p.7 the authors describe that the interval between sessions depended on school arrangement and availability of subjects and that it could vary between 1 hour and 2 days. This large variation

is not further discussed. Could this large interval variation have had an effect on the result? The authors write that it was typically around 2 hours. I believe a more precise measure of interval variation ( M and SD) would be meaningful in order evaluate its possible effect . Also, the testing seem to have been conducted by three different experimental leaders (RM AF and ND). Did the authors test for a possible test leader effect? If not, Please check this.

Data Analysis and Results

No information is given regarding outliers, normality tests etc. Please add.
A very important point is that no descriptive data (M , SD; min, max for the different age groups for all of the tasks) is provided neither in the manuscript nor in the supplementary analysis. Such data is key for interpreting the results especially regarding the executive function measures and their relation to the flexible planning task. For example, were there any floor or ceiling effects for the EF tasks? For example, there were no age effects on the knock-tap task. Could it be that this lack of age effect is due to floor or ceiling effects in these children? Please add descriptive data for all the tasks included in the study.

Discussion

Overall, from my perspective, the take home message of this study is not clearly conveyed in the discussion. What is really the key finding from this study? And how does it contribute novel knowledge to the field. Please state this essential information clearly in the beginning as well as at the end of the discussion ( as well as in the abstract). Similarly, to the introduction, I think that the discussion would gain from reorganizing/restructuring in relation to aims and key findings. In its current form, the reasoning is hard to follow and gives a jumbled impression.
Here are some other points that I am not clear about in the discussion/ interpretation of the results.

To me, it was surprising that the EF measures were significantly correlated with one another ( please remove the word "highly" from this sentence (p.13) as the effect size of these correlations cannot be considered as strong/high (Ref Cohen ) as this is certainly not always the case with young children. Considering that EF is a non-unitary construct with different developmental trajectories for the different components, I think it would have been interesting to see how the EF tasks related to each other within the three age groups.

It was interesting to note that the only EF measure that correlated with the flexible planning task was the knock tap task, which was also the only EF measure that did not show age effects. How does this fact influence the interpretation of the result? Again, here it is crucial to know the descriptive data in order to make a more complete interpretation of the observed relation between these measures. Since the knock and tap task taxes the same type of complex inhibition as the day/night task ( keep a rule in mind as well as inhibiting the most spontaneous response in favor of the correct response) except for the verbal component in the day/night task, makes me wonder what this relation really stands for. It may be non-executive, perhaps motoric, in nature. The authors do touch upon this reasoning, but I think it could be spelled out more clearly. Having said this, I am not really sure what the authors mean by saying that the lack of correlation with our other inhibitory controls measure ( day/night task) indicate that there does not appear to be a "general difficulty" with inhibition influencing flexible planning performance. As mentioned above, both inhibitory tasks measure the same type of complex inhibition and one but not the other related to the planning task. Thus, I do not believe that this conclusion can be drawn based on the data. Also, the reasoning around the relations between executive function and flexible planning on p.14 appears quite weak, e.g., "Inhibition may correlate with performance in some flexible planning task" –please elaborate on type of inhibition and what aspect of the planning that is referred to.
Since the correlational data is not included in the manuscript, it is important that the direction of the relationships are clearly expressed in the discussion. Please add this information.

Limitations of this study should be stated. Please add (e.g., the study being cross-sectional). No measure of episodic memory.
Finally, the task is suggested to be of use for research on this topic in other species. I am not convinced that this would actually work (what species did the authors have in mind more precisely). Please elaborate.

# Decision letter (RSOS-190894.R0)

22-Jul-2019

Dear Dr Frohnwieser:

Manuscript ID RSOS-190894 entitled "Executive function, language and flexible planning in young children" which you submitted to Royal Society Open Science, has been reviewed. The comments from reviewers are included at the bottom of this letter.

In view of the criticisms of the reviewers, the manuscript has been rejected in its current form. However, a new manuscript may be submitted which takes into consideration these comments.

Please note that resubmitting your manuscript does not guarantee eventual acceptance, and that your resubmission will be subject to peer review before a decision is made.

You will be unable to make your revisions on the originally submitted version of your manuscript. Instead, revise your manuscript and upload the files via your author centre.

Once you have revised your manuscript, go to https://mc.manuscriptcentral.com/rsos and login to your Author Center. Click on "Manuscripts with Decisions," and then click on "Create a Resubmission" located next to the manuscript number. Then, follow the steps for resubmitting your manuscript.

Your resubmitted manuscript should be submitted by 19-Jan-2020. If you are unable to submit by this date please contact the Editorial Office.

We look forward to receiving your resubmission.

Kind regards,
Andrew Dunn
Royal Society Open Science Editorial Office
Royal Society Open Science
openscience@royalsociety.org

on behalf of Dr Teodora Gliga (Associate Editor) and Essi Viding (Subject Editor)
openscience@royalsociety.org

Associate Editor Comments to Author (Dr Teodora Gliga):
Associate Editor: 1
Comments to the Author:
Dear Dr. Frohnwieser,

I have received now reviews from two experts in the field and both have raised major concerns with respect to the data collection (e.g. more information needed regarding the timing of the

tasks), data analysis (e.g. corrections for multiple comparisons) and interpretation (e.g. what do the tasks measure, what the findings imply in terms of underlying mechanisms).

I will therefore reject the manuscript at this point but allow a resubmission. You have up to 6 months to complete a resubmission.

Reviewers' Comments to Author:
Reviewer: 1

Comments to the Author(s)
Summary
This is an interesting study, using innovative and age-appropriate tasks, which will be of particular value to researchers interested in using common measures in comparative research with human and non-human animals. Given the current emphasis in the paper on the association between flexible planning and EF, I recommend that that the statistical tests used are corrected for multiple comparisons and the discussion reframed accordingly.

Major comments
1. SM Table 5 (and all related discussion and conclusions): Correction should be made for multiple comparisons – and the discussion adjusted accordingly. Knock-tap does not correlate with flexible planning performance within any of the age bands; this should be referred to in discussion.

2. Please present plots of the key correlations referred to in the discussion (i.e. Knock-tap with flexible planning). Do ceiling effects in the planning task influence the association?

3. Lines 196-200: In the explanation of the Session 1 (pre-experience phase) it is not clear to me how the most- and least-preferred stickers are defined. If the child selects the (researcher-defined) 'low quality' white sticker, does that make it the most-preferred option for that child? If not, why not? If the child does not show a preference for the 'high quality' sticker is that accounted for in step 3?

4. The variable labels in the supporting data do not make it clear which is the variable used for 'flexible planning' (in Supplementary table 5); please clarify. Further, I have not been able to reproduce the correlation matrix presented in Supplementary table 5 and sample sizes vary from that presented (e.g. in the supporting data provided, n=86 participants contributed knock-tap data and forward digit span data, but only n=84 are presented in Supplementary table 5.

Minor comments

5. Line 147. Please provide a justification (and references) for your characterisation of the different tasks as distinctly capturing inhibitory control, cognitive flexibility and working memory

6. Line 189. Please present the number of children with an inter-session interval greater than two hours. It seems likely that the inter-session interval might influence performance – please elaborate on this in the supplementary materials.

7. Line 201: I am not convinced that step 3 of the session 1 procedure can truly be characterised as delay of gratification if, as is indicated in the discussion on p12 line 365, children find tool use gratifying in itself. Please justify this characterisation or reframe step 3. Related to this, it might be informative for future studies (not expected here) to have a condition in which the kit is not baited; do participants still drop the stone in just because it is fun?

8. I would find it useful to have the correlations currently presented in Supplementary Table 5 within the main manuscript. The table would also benefit from clearer formatting/layout.

9. The correlation between flexible planning and language ability has not been corrected for multiple tests) and may be a false positive; this should be considered in the discussion

10. Lines 354-356: I think that the statement that understanding of 'yesterday' and 'tomorrow' is not yet established in 3 year olds is an overly broad assertion if based only on the Harner study cited (which features just 30 3-year-olds, with 1 paradigm, and does not report the proportions of 3-year-olds who do and do not understand these concepts). Please provide further support for this statement (or omit it from the discussion).

11. Lines 360-361: The statement that 'children of all ages were able to inhibit the selection of an immediately available low-value reward for a tool in order to obtain a higher value reward inside the apparatus' needs some unpacking/reframing. There is an implication here that there was an objectively better sticker; returning to my previous comment re line 196-200 what if the child preferred white stickers? Secondly, an inhibitory demand would be expect to lead to a performance cost yet participants performed better when the high-value reward was inside the apparatus. Thirdly, in my view the lack of association between the day/night task (which is described here as an inhibitory control measure) and flexible planning performance indicates that the demands of the task are not primarily inhibitory.

12. Lines 383-384: This sentence is self-contradictory; EF is either unitary or has 3 components. But either way I am not convinced that the literature currently supports a strong claim in either direction. The Miyake et al. (2000) paper was followed by a study by Miyake and Friedman (2012) demonstrating that, in adolescents and young adults, two dissociable EF components can be identified, as well as a 'common EF' component that is shared across tasks (and it should be noted, that this model is specific to the tasks used; the authors acknowledge that different tasks might yield different conclusions). Amongst pre-schoolers it has been argued that a single latent EF construct best describes pre-schoolers' performance on EF batteries (Hughes, Ensor, Wilson, & Graham, 2009; Senn, Espy, & Kaufmann, 2004; Wiebe, Espy, & Charak, 2008; Wiebe et al., 2011; Willoughby, Wirth, Blair & Family Life Project Investigators, 2012), and that EF factors can be dissociated (Bernier, Carlson, Deschênes, & Matte-Gagné, 2012; Garon, Smith, & Bryson, 2014; Mulder, Hoofs, Verhagen, van der Veen, & Leseman, 2014; Skogan et al., 2015).

13. Line 393: It should be clarified that in this study one 1 inhibition task correlated with flexible planning performance

Reviewer: 2

Comments to the Author(s)
Comments to the Author

This is my first review of the manuscript entitled "Executive function, language and flexible planning in young children."
This cross-sectional research builds on a new experimental tool-use paradigm designed to assess flexible planning in 3 to 5 year olds. The task is designed to address particular flaws that have been observed in earlier studies on this topic. Particularly, single trials were used to avoid repeated exposure to stimulus-reward relationships, training trials provided the participants with experience of a predictable return of reward, children were not verbally cued during the task, and delays were included before and after tool choice. Relations between flexible planning performance in this paradigm and receptive language as well as executive functioning were also examined. See comments to authors below.

Title: My understanding is that the flexible planning is the main part of this study and therefore I think it would be more descriptive with a title like, Flexible planning in 3-5 year old children and relations

Introduction

The introduction is relatively well-written, but would gain from a re-write/structure in relation to the order of the aims of this study, which appears to be several and somewhat unclear and unstructured. I would recommend the authors to think through what the main, second and third aim etc of this study really are and structure the introduction in the same order. To my understanding the following aspects are included in aims: to design a task that can be comparable with non-human studies, to design a task that addresses flaws in earlier paradigms on the same topic ( see paragraph above), to examine the role of delay of gratification in flexible planning, to examine relations with language and several executive functions. Please order these according to their significance and re-structure the introduction correspondingly.
Surprisingly, there are no predictions/ hypotheses formulated regarding the research questions asked. Please add predictions in relation to each research questions under the aims section.
A minor ( yet important) point in the introduction ( and discussion) is that the authors describe executive functions as a unitary function. As is empirically established by Miyake and others ( who the authors also refer to) EF is a complex and very broad higher order cognitive construct that show both unity and diversity and should be referred to accordingly. Please change.

Methods

Subjects. The sample should be described more thoroughly. What was the inclusion/exclusion criteria? Were they all typically developing? Born after week 36? Etc. Measures of SES should be included ( for example; income and parents 'education level).
Apparatus. Although the supplementary movie and figures were helpful in understanding the apparatus and procedure. Indeed, the apparatus and procedure used is quite complex and the authors need to make sure that it is clearly described in the manuscript to ease the understanding of the readers. For example, make sure to describe the three different apparatuses used in the same or that they are illustrated in Fig 1. Or if described in the same order as they were used, please change order of picture accordingly. I had a hard time understanding the apparatus and the different steps in the procedure and had to read these sections many times. Please think through how this section can be described even more clearly, so the reader can understand it without having to watch the movie.

Procedure

On p.7 the authors describe that the interval between sessions depended on school arrangement and availability of subjects and that it could vary between 1 hour and 2 days. This large variation is not further discussed. Could this large interval variation have had an effect on the result? The authors write that it was typically around 2 hours. I believe a more precise measure of interval variation ( M and SD) would be meaningful in order evaluate its possible effect . Also, the testing seem to have been conducted by three different experimental leaders (RM AF and ND). Did the authors test for a possible test leader effect? If not, Please check this.

Data Analysis and Results

No information is given regarding outliers, normality tests etc. Please add.
A very important point is that no descriptive data (M , SD; min, max for the different age groups for all of the tasks) is provided neither in the manuscript nor in the supplementary analysis. Such data is key for interpreting the results especially regarding the executive function measures and their relation to the flexible planning task. For example, were there any floor or ceiling effects for the EF tasks? For example, there were no age effects on the knock-tap task. Could it be that this lack of age effect is due to floor or ceiling effects in these children? Please add descriptive data for all the tasks included in the study.

Discussion

Overall, from my perspective, the take home message of this study is not clearly conveyed in the discussion. What is really the key finding from this study? And how does it contribute novel knowledge to the field. Please state this essential information clearly in the beginning as well as at the end of the discussion ( as well as in the abstract). Similarly, to the introduction, I think that the discussion would gain from reorganizing/restructuring in relation to aims and key findings. In its current form, the reasoning is hard to follow and gives a jumbled impression.
Here are some other points that I am not clear about in the discussion/ interpretation of the results.

To me, it was surprising that the EF measures were significantly correlated with one another ( please remove the word "highly" from this sentence (p.13) as the effect size of these correlations cannot be considered as strong/high (Ref Cohen ) as this is certainly not always the case with young children. Considering that EF is a non-unitary construct with different developmental trajectories for the different components, I think it would have been interesting to see how the EF tasks related to each other within the three age groups.

It was interesting to note that the only EF measure that correlated with the flexible planning task was the knock tap task, which was also the only EF measure that did not show age effects. How does this fact influence the interpretation of the result? Again, here it is crucial to know the descriptive data in order to make a more complete interpretation of the observed relation between these measures. Since the knock and tap task taxes the same type of complex inhibition as the day/night task ( keep a rule in mind as well as inhibiting the most spontaneous response in favor of the correct response) except for the verbal component in the day/night task, makes me wonder what this relation really stands for. It may be non-executive, perhaps motoric, in nature. The authors do touch upon this reasoning, but I think it could be spelled out more clearly. Having said this, I am not really sure what the authors mean by saying that the lack of correlation with our other inhibitory controls measure ( day/night task) indicate that there does not appear to be a "general difficulty" with inhibition influencing flexible planning performance. As mentioned above, both inhibitory tasks measure the same type of complex inhibition and one but not the other related to the planning task. Thus, I do not believe that this conclusion can be drawn based on the data. Also, the reasoning around the relations between executive function and flexible planning on p.14 appears quite weak, e.g., "Inhibition may correlate with performance in some flexible planning task" –please elaborate on type of inhibition and what aspect of the planning that is referred to.
Since the correlational data is not included in the manuscript, it is important that the direction of the relationships are clearly expressed in the discussion. Please add this information.
Limitations of this study should be stated. Please add (e.g., the study being cross-sectional). No measure of episodic memory.
Finally, the task is suggested to be of use for research on this topic in other species. I am not convinced that this would actually work (what species did the authors have in mind more precisely). Please elaborate.

# Author's Response to Decision Letter for (RSOS-190894.R0)

See Appendix A.

# RSOS-192015.R0

## Review form: Reviewer 1

**Is the manuscript scientifically sound in its present form?**
Yes

**Are the interpretations and conclusions justified by the results?**
Yes

**Is the language acceptable?**
Yes

**Do you have any ethical concerns with this paper?**
No

**Have you any concerns about statistical analyses in this paper?**
No

**Recommendation?**
Accept with minor revision (please list in comments)

**Comments to the Author(s)**
The authors have responded appropriately to the previous round of comments and this manuscript provides a well-reasoned explanation of the study design and results. I have some further minor amends to suggest, below, but no major concerns with the manuscript as it currently stands.

Minor amends
1. Given that the authors concede that the tool is gratifying so the sticker-tool choice doesn't actually work as a delay of gratification task, the sentence
First, we tested delay of gratification by asking children to choose between an immediately available reward of lower quality or a tool to obtain a delayed reward of higher quality
Should be amended to
First, we aimed to test delay of gratification by asking children to choose between an immediately available reward of lower quality or a tool to obtain a delayed reward of higher quality

2. Add to the limitations an acknowledgement that this (presumably) was not a diverse sample, drawn only from a British sample and with no monitoring or attempt to diversity SES
3. Add to the limitations section that 7 out of 87 children were tested on a different day to the training session
4. Add to the Methods section that participants are presumed to be typically-developing but that no screening was attempted
5. In the sentence "Adopting factorial analytic approach, researchers have identified three key components of executive factors; inhibitory control, working memory and cognitive flexibility" clarify that this is in adults
6. The sentence "Executive function is a unitary construct with three core components: inhibition, working memory and cognitive shifting/flexibility" is not justified by the arguments above. It is possible, on the basis of the 2 studies mentioned, that the nature of executive function changes across development, from a primarily inhibitory ('Common EF') function, to a more complex, multi-component function in later development. Further there are other influential theoretical models of EF and the Miyake model only strictly applies to the population and tasks used in that study. To allow for other models, the framing should be something like 'Influenced by the work of Miyake et al which showed that ….. along with developmental research indicating that in

young children EF may be a more unitary construct driven primarily by inhibitory control… we have selected…..”
7. Clarify the expected direction of the correlations (last sentence before the methods)
8. Add details of the EF task administration protocols in the Supplementary materials, and references to the original sources
9. In the methods section, indicate which EF task is designed to index which component skill
10. The following sentence seems incomplete “For example, corvids (members of the crow family) have shown impressive flexible planning skills in a caching, for example in learning what to cache and what not to cache based on the foods available at the time of cache recovery “
11. Should Subjects not be participants throughout?
12. It is not clear to me that the logic of the following sentence holds true: “Finally, as the children performed similarly in our flexible planning task as in previous developmental studies, we feel our new paradigm provides an exciting opportunity to further explore episodic foresight across different species, specifically corvids, such as tool-using Caledonian crows, or primates”. Why should showing a developmental improvement in children mean the task could be used in non-human animals? Please make this argument clearer, or drop. This point also applies to the very last sentence.

## Review form: Reviewer 2

**Is the manuscript scientifically sound in its present form?**
Yes

**Are the interpretations and conclusions justified by the results?**
Yes

**Is the language acceptable?**
Yes

**Do you have any ethical concerns with this paper?**
No

**Have you any concerns about statistical analyses in this paper?**
No

**Recommendation?**
Major revision is needed (please make suggestions in comments)

**Comments to the Author(s)**
The authors have done a pretty good job with addressing most of my comments in the previous version of this manuscript.
However, I am quite concerned about the fact that the main/or overall aim of this study is still not clear. Is it to validate this new task of flexible planning by looking at expected age effects and in addition investigating relations to executive function performance and language as part of this validation? In the aim section at the end of the introduction the main aim is described as “just” studying correlations between this new task and executive functions and language, whereas in the discussion it appears as the relations between different functions is a secondary aim. Further, the title says nothing about that a novel task of flexible planning is explored. Again, I think the authors really need to think through what the main aim really is and the clearly and consistently express this throughout the manuscript.

Also, the sentence now describing the main aim (line 248) begins by saying Our third aim – please delete third here. Further, analyses for the relations between flexible planning and

executive functions and language are provided per age group, but predictions are only included for the total sample regarding these relations. Do the authors expect age effects in these relations? Please add predictions regarding the relations per age group.

Minor: it appears that executive function on p.17 is still described as unitary construct. If the authors mean that previous findings show that the construct seem to load on a single factor in early development and in the preschool year this should be more clearly expressed. Also there seem to be reiteration between the sentence on line 183-187 and the one on line 192-198. I suggest the last part of the first sentence ( 183-187) -"yet preschoolers performance on various tasks can be explained by a single theoretical latent factor" should be deleted. By the way, it is unclear to me what a theoretical factor here means.

I

# Decision letter (RSOS-192015.R0)

21-Feb-2020

Dear Dr Frohnwieser

On behalf of the Editor, I am pleased to inform you that your Manuscript RSOS-192015 entitled "Flexible planning, executive function and language in young children" has been accepted for publication in Royal Society Open Science subject to minor revision in accordance with the referee suggestions. Please find the referees' comments at the end of this email.

The reviewers and Subject Editor have recommended publication, but also suggest some minor revisions to your manuscript. Therefore, I invite you to respond to the comments and revise your manuscript.

• Ethics statement
If your study uses humans or animals please include details of the ethical approval received, including the name of the committee that granted approval. For human studies please also detail whether informed consent was obtained. For field studies on animals please include details of all permissions, licences and/or approvals granted to carry out the fieldwork.

• Data accessibility
It is a condition of publication that all supporting data are made available either as supplementary information or preferably in a suitable permanent repository. The data accessibility section should state where the article's supporting data can be accessed. This section should also include details, where possible of where to access other relevant research materials such as statistical tools, protocols, software etc can be accessed. If the data has been deposited in an external repository this section should list the database, accession number and link to the DOI for all data from the article that has been made publicly available. Data sets that have been deposited in an external repository and have a DOI should also be appropriately cited in the manuscript and included in the reference list.

If you wish to submit your supporting data or code to Dryad (http://datadryad.org/), or modify your current submission to dryad, please use the following link:
http://datadryad.org/submit?journalID=RSOS&manu=RSOS-192015

• Competing interests
Please declare any financial or non-financial competing interests, or state that you have no competing interests.

• Authors' contributions
All submissions, other than those with a single author, must include an Authors' Contributions section which individually lists the specific contribution of each author. The list of Authors should meet all of the following criteria; 1) substantial contributions to conception and design, or acquisition of data, or analysis and interpretation of data; 2) drafting the article or revising it critically for important intellectual content; and 3) final approval of the version to be published.

All contributors who do not meet all of these criteria should be included in the acknowledgements.

We suggest the following format:
AB carried out the molecular lab work, participated in data analysis, carried out sequence alignments, participated in the design of the study and drafted the manuscript; CD carried out the statistical analyses; EF collected field data; GH conceived of the study, designed the study, coordinated the study and helped draft the manuscript. All authors gave final approval for publication.

• Acknowledgements
Please acknowledge anyone who contributed to the study but did not meet the authorship criteria.

• Funding statement
Please list the source of funding for each author.

Please note that we cannot publish your manuscript without these end statements included. We have included a screenshot example of the end statements for reference. If you feel that a given heading is not relevant to your paper, please nevertheless include the heading and explicitly state that it is not relevant to your work.

Because the schedule for publication is very tight, it is a condition of publication that you submit the revised version of your manuscript before 01-Mar-2020. Please note that the revision deadline will expire at 00.00am on this date. If you do not think you will be able to meet this date please let me know immediately.

To revise your manuscript, log into https://mc.manuscriptcentral.com/rsos and enter your Author Centre, where you will find your manuscript title listed under "Manuscripts with Decisions". Under "Actions," click on "Create a Revision." You will be unable to make your revisions on the originally submitted version of the manuscript. Instead, revise your manuscript and upload a new version through your Author Centre.

When submitting your revised manuscript, you will be able to respond to the comments made by the referees and upload a file "Response to Referees" in "Section 6 - File Upload". You can use this to document any changes you make to the original manuscript. In order to expedite the processing of the revised manuscript, please be as specific as possible in your response to the referees.

When uploading your revised files please make sure that you have:

1) A text file of the manuscript (tex, txt, rtf, docx or doc), references, tables (including captions) and figure captions. Do not upload a PDF as your "Main Document".
2) A separate electronic file of each figure (EPS or print-quality PDF preferred (either format should be produced directly from original creation package), or original software format)
3) Included a 100 word media summary of your paper when requested at submission. Please ensure you have entered correct contact details (email, institution and telephone) in your user account

4) Included the raw data to support the claims made in your paper. You can either include your data as electronic supplementary material or upload to a repository and include the relevant doi within your manuscript
5) All supplementary materials accompanying an accepted article will be treated as in their final form. Note that the Royal Society will neither edit nor typeset supplementary material and it will be hosted as provided. Please ensure that the supplementary material includes the paper details where possible (authors, article title, journal name).

Supplementary files will be published alongside the paper on the journal website and posted on the online figshare repository (https://figshare.com). The heading and legend provided for each supplementary file during the submission process will be used to create the figshare page, so please ensure these are accurate and informative so that your files can be found in searches. Files on figshare will be made available approximately one week before the accompanying article so that the supplementary material can be attributed a unique DOI.

Once again, thank you for submitting your manuscript to Royal Society Open Science and I look forward to receiving your revision. If you have any questions at all, please do not hesitate to get in touch.

Kind regards,
Andrew Dunn
Royal Society Open Science Editorial Office
Royal Society Open Science
openscience@royalsociety.org

on behalf of Dr Teodora Gliga (Associate Editor) and Essi Viding (Subject Editor)
openscience@royalsociety.org

Associate Editor Comments to Author (Dr Teodora Gliga):
Associate Editor
Comments to the Author:
Your revision was now seen by the two original reviewers. Although they both indicate that you have addressed many of their comments, there remain some inconsistencies in the way you describe the literature or the findings, that you need to address. Most importantly, I urge you to clearly state the aims of the study and make sure these are consistently indicated throughout the manuscript (title, abstract, main text).

Reviewer comments to Author:
Reviewer: 1

Comments to the Author(s)
The authors have responded appropriately to the previous round of comments and this manuscript provides a well-reasoned explanation of the study design and results. I have some further minor amends to suggest, below, but no major concerns with the manuscript as it currently stands.

Minor amends
1. Given that the authors concede that the tool is gratifying so the sticker-tool choice doesn't actually work as a delay of gratification task, the sentence
First, we tested delay of gratification by asking children to choose between an immediately available reward of lower quality or a tool to obtain a delayed reward of higher quality
Should be amended to
First, we aimed to test delay of gratification by asking children to choose between an immediately available reward of lower quality or a tool to obtain a delayed reward of higher quality

2. Add to the limitations an acknowledgement that this (presumably) was not a diverse sample, drawn only from a British sample and with no monitoring or attempt to diversity SES
3. Add to the limitations section that 7 out of 87 children were tested on a different day to the training session
4. Add to the Methods section that participants are presumed to be typically-developing but that no screening was attempted
5. In the sentence "Adopting factorial analytic approach, researchers have identified three key components of executive factors; inhibitory control, working memory and cognitive flexibility" clarify that this is in adults
6. The sentence "Executive function is a unitary construct with three core components: inhibition, working memory and cognitive shifting/flexibility" is not justified by the arguments above. It is possible, on the basis of the 2 studies mentioned, that the nature of executive function changes across development, from a primarily inhibitory ('Common EF') function, to a more complex, multi-component function in later development. Further there are other influential theoretical models of EF and the Miyake model only strictly applies to the population and tasks used in that study. To allow for other models, the framing should be something like 'Influenced by the work of Miyake et al which showed that ….. along with developmental research indicating that in young children EF may be a more unitary construct driven primarily by inhibitory control… we have selected….."
7. Clarify the expected direction of the correlations (last sentence before the methods)
8. Add details of the EF task administration protocols in the Supplementary materials, and references to the original sources
9. In the methods section, indicate which EF task is designed to index which component skill
10. The following sentence seems incomplete "For example, corvids (members of the crow family) have shown impressive flexible planning skills in a caching, for example in learning what to cache and what not to cache based on the foods available at the time of cache recovery "
11. Should Subjects not be participants throughout?
12. It is not clear to me that the logic of the following sentence holds true: "Finally, as the children performed similarly in our flexible planning task as in previous developmental studies, we feel our new paradigm provides an exciting opportunity to further explore episodic foresight across different species, specifically corvids, such as tool-using Caledonian crows, or primates". Why should showing a developmental improvement in children mean the task could be used in non-human animals? Please make this argument clearer, or drop. This point also applies to the very last sentence.

Reviewer: 2

Comments to the Author(s)
The authors have done a pretty good job with addressing most of my comments in the previous version of this manuscript.
However, I am quite concerned about the fact that the main/or overall aim of this study is still not clear. Is it to validate this new task of flexible planning by looking at expected age effects and in addition investigating relations to executive function performance and language as part of this validation? In the aim section at the end of the introduction the main aim is described as "just" studying correlations between this new task and executive functions and language, whereas in the discussion it appears as the relations between different functions is a secondary aim. Further, the title says nothing about that a novel task of flexible planning is explored. Again, I think the authors really need to think through what the main aim really is and the clearly and consistently express this throughout the manuscript.

Also, the sentence now describing the main aim (line 248) begins by saying Our third aim – please delete third here. Further, analyses for the relations between flexible planning and executive functions and language are provided per age group, but predictions are only included

for the total sample regarding these relations. Do the authors expect age effects in these relations? Please add predictions regarding the relations per age group.

Minor: it appears that executive function on p.17 is still described as unitary construct. If the authors mean that previous findings show that the construct seem to load on a single factor in early development and in the preschool year this should be more clearly expressed. Also there seem to be reiteration between the sentence on line 183-187 and the one on line 192-198. I suggest the last part of the first sentence ( 183-187) -"yet preschoolers performance on various tasks can be explained by a single theoretical latent factor" should be deleted. By the way, it is unclear to me what a theoretical factor here means.

I

## Author's Response to Decision Letter for (RSOS-192015.R0)

See Appendix B.

## Decision letter (RSOS-192015.R1)

10-Mar-2020

Dear Dr Frohnwieser,

It is a pleasure to accept your manuscript entitled "A novel test of flexible planning in relation to executive function and language in young children" in its current form for publication in Royal Society Open Science.

In addition, we note that, firstly, saj48@cam.ac.uk is not currently accepting emails from Royal Society Open Science - please can you ensure you supply the editorial office with an active email address for your colleague?

Secondly, you had previously requested that Dr Miller be set as the corresponding author for the manuscript - please can I confirm that this should be the case for all production queries and on the final published version of the paper?

You can expect to receive a proof of your article in the near future. Please contact the editorial office (openscience_proofs@royalsociety.org) and the production office (openscience@royalsociety.org) to let us know if you are likely to be away from e-mail contact -- if you are going to be away, please nominate a co-author (if available) to manage the proofing process, and ensure they are copied into your email to the journal.

Due to rapid publication and an extremely tight schedule, if comments are not received, your paper may experience a delay in publication.

Please see the Royal Society Publishing guidance on how you may share your accepted author manuscript at https://royalsociety.org/journals/ethics-policies/media-embargo/.

Thank you for your fine contribution. On behalf of the Editors of Royal Society Open Science, we look forward to your continued contributions to the Journal.

Kind regards,
Andrew Dunn
Royal Society Open Science Editorial Office
Royal Society Open Science
openscience@royalsociety.org

on behalf of Dr Teodora Gliga (Associate Editor) and Essi Viding (Subject Editor)
openscience@royalsociety.org

Follow Royal Society Publishing on Twitter: @RSocPublishing
Follow Royal Society Publishing on Facebook:
https://www.facebook.com/RoyalSocietyPublishing.FanPage/
Read Royal Society Publishing's blog: https://blogs.royalsociety.org/publishing/

# Appendix A

Dear Mr Dunn and Dr Gliga,

Following an invitation to revise and re-submit, we would like to re-submit our manuscript ID RSOS-190894 entitled "Executive function, language and flexible planning in young children".

We wish to thank the two reviewers for the helpful and constructive comments. We have now fully revised the manuscript and accompanying documents in accordance with these comments. Please find responses to each comment in this response to reviewers document .

We hope that following our revisions, you will consider our manuscript for publication in Royal Society Open Science.

Yours Sincerely,

Rachael Miller, Anna Frohnwieser, Ning Ding, Camille Troisi, Martina Schiestl, Romana Gruber, Alex Taylor, Sarah Jelbert, Markus Boeckle and Nicky Clayton

Dear Dr Frohnwieser:

Manuscript ID RSOS-190894 entitled "Executive function, language and flexible planning in young children" which you submitted to Royal Society Open Science, has been reviewed. The comments from reviewers are included at the bottom of this letter.

In view of the criticisms of the reviewers, the manuscript has been rejected in its current form. However, a new manuscript may be submitted which takes into consideration these comments.

Please note that resubmitting your manuscript does not guarantee eventual acceptance, and that your resubmission will be subject to peer review before a decision is made.

You will be unable to make your revisions on the originally submitted version of your manuscript. Instead, revise your manuscript and upload the files via your author centre.

Once you have revised your manuscript, go to https://mc.manuscriptcentral.com/rsos and login to your Author Center. Click on "Manuscripts with Decisions," and then click on "Create a Resubmission" located next to the manuscript number. Then, follow the steps for resubmitting your manuscript.

Your resubmitted manuscript should be submitted by 19-Jan-2020. If you are unable to submit by this date please contact the Editorial Office.

We look forward to receiving your resubmission.

Kind regards,
Andrew Dunn
Royal Society Open Science Editorial Office
Royal Society Open Science

openscience@royalsociety.org

on behalf of Dr Teodora Gliga (Associate Editor) and Essi Viding (Subject Editor)
openscience@royalsociety.org

Associate Editor Comments to Author (Dr Teodora Gliga):
Associate Editor: 1
Comments to the Author:
Dear Dr. Frohnwieser,

I have received now reviews from two experts in the field and both have raised major concerns with respect to the data collection (e.g. more information needed regarding the timing of the tasks), data analysis (e.g. corrections for multiple comparisons) and interpretation (e.g. what do the tasks measure, what the findings imply in terms of underlying mechanisms).

I will therefore reject the manuscript at this point but allow a resubmission. You have up to 6 months to complete a resubmission.

Reviewers' Comments to Author:
**Reviewer: 1**

Comments to the Author(s)
Summary
This is an interesting study, using innovative and age-appropriate tasks, which will be of particular value to researchers interested in using common measures in comparative research with human and non-human animals. Given the current emphasis in the paper on the association between flexible planning and EF, I recommend that that the statistical tests used are corrected for multiple comparisons and the discussion reframed accordingly.

**Thank you for your comment**

Major comments
1. SM Table 5 (and all related discussion and conclusions): Correction should be made for multiple comparisons – and the discussion adjusted accordingly. Knock-tap does not correlate with flexible planning performance within any of the age bands; this should be referred to in discussion.

**A Bonferonni correction was applied to the correlation matrix to correct for multiple comparisons. This changed the results, so that none of the executive function tasks correlate with performance in the flexible planning test. The results, discussion and abstract have been adjusted accordingly.**

2. Please present plots of the key correlations referred to in the discussion (i.e. Knock-tap with flexible planning). Do ceiling effects in the planning task influence the association?

**A table indicating the mean performance ± standard deviation for each test and age group was added (Table 3). This shows that there were no ceiling effects for any of the tasks in any of the age groups.**

3. Lines 196-200: In the explanation of the Session 1 (pre-experience phase) it is not clear to me how the most- and least-preferred stickers are defined. If the child selects the (researcher-defined) 'low quality' white sticker, does that make it the most-preferred option for that child? If not, why not? If the child does not show a preference for the 'high quality' sticker is that accounted for in step 3?

**We found that all children had a preference for the high-quality stickers, which had colourful pictures of animals, over the plain white stickers. We clarified this in the description of Session 1 (line 213-214).**

4. The variable labels in the supporting data do not make it clear which is the variable used for 'flexible planning' (in Supplementary table 5); please clarify.

**The variable labels in the main manuscript, supporting data and Figshare data file have been adjusted to correspond across all files.**

Further, I have not been able to reproduce the correlation matrix presented in Supplementary table 5

**Thank you. The results and discussion section have been adjusted accordingly.**

and sample sizes vary from that presented (e.g. in the supporting data provided, n=86 participants contributed knock-tap data and forward digit span data, but only n=84 are presented in Supplementary table 5.

**There are n=86 participants that contributed to the forward digit span data, but only n=84 that contributed to the knock-tap data, so the sample size for the correlation is n=84.**

Minor comments

5. Line 147. Please provide a justification (and references) for your characterisation of the different tasks as distinctly capturing inhibitory control, cognitive flexibility and working memory

**Miyake's influential work with adults identified three core factors within the central executive function, inhibition, working memory and shifting. Executive function can be considered as a unitary construct with partially dissociable components (Garon, Bryson & Smith, 2008; Miyake et al., 2000; Miyake & Freeman, 2012). This has laid the foundation for various measurements of executive function (Carlson, 2005). We have added a paragraph in the introduction explaining this characterisation (line 113-126).**

6. Line 189. Please present the number of children with an inter-session interval greater than two hours. It seems likely that the inter-session interval might influence performance – please elaborate on this in the supplementary materials.

**There were 7 children (out of 87) who received the test session on a different day than the training session. Due to this small number, it was not possible to determine whether this had an influence on their performance. We have now clarified this in the "Procedure" section (line 200-202).**

7. Line 201: I am not convinced that step 3 of the session 1 procedure can truly be characterised as delay of gratification if, as is indicated in the discussion on p12 line 365, children find tool use gratifying in itself. Please justify this characterisation or reframe step 3. Related to this, it might be informative for future studies (not expected here) to have a condition in which the kit is not baited; do participants still drop the stone in just because it is fun?

**We agree that tool use itself seems to have been rewarding for the children, making it questionable if step 3 actually measured delay of gratification. We have added a discussion of this in the discussion section (line 422-427). However, as we originally added step 3 with the intent to give the children experience with delay of gratification and only after testing found that children performed better when being able to use tools, we do not feel comfortable changing this description in the methods section.**

8. I would find it useful to have the correlations currently presented in Supplementary Table 5 within the main manuscript. The table would also benefit from clearer formatting/layout.

**Supplementary Table 5 was moved to the results section of the main manuscript (Table 4) and the variable names have been changed to clarify which tasks they are referring to.**

9. The correlation between flexible planning and language ability has not been corrected for multiple tests) and may be a false positive; this should be considered in the discussion

**A Bonferonni correction was applied to the correlation matrix, and results and discussion adjusted accordingly.**

10. Lines 354-356: I think that the statement that understanding of 'yesterday' and 'tomorrow' is not yet established in 3 year olds is an overly broad assertion if based only on the Harner study cited (which features just 30 3-year-olds, with 1 paradigm, and does not report the proportions of 3-year-olds who do and do not understand these concepts). Please provide further support for this statement (or omit it from the discussion).

**As some of our results have changed due to a new analysis, this section has been removed.**

11. Lines 360-361: The statement that 'children of all ages were able to inhibit the selection of an immediately available low-value reward for a tool in order to obtain a higher value reward inside the apparatus' needs some unpacking/reframing. There is an implication here that there was an objectively better sticker; returning to my previous comment re line 196-200 what if the child preferred white stickers?

**All children in our experiment preferred the colourful sticker over the white sticker, which was confirmed by preference tests. This information has also been added to the methods section (line 213-214).**

Secondly, an inhibitory demand would be expect to lead to a performance cost yet participants performed better when the high-value reward was inside the apparatus.

**We agree that it seems like tool use itself was rewarding for the subjects, and therefore trials in which the choice was between an immediate, low quality reward and a tool to obtain a higher quality reward may not truly have measured delay of gratification. We have now added a discussion of this (line 422-427).**

Thirdly, in my view the lack of association between the day/night task (which is described here as an inhibitory control measure) and flexible planning performance indicates that the demands of the task are not primarily inhibitory.

**As our results have changed, we have addressed these in the discussion (line 388-417).**

12. Lines 383-384: This sentence is self-contradictory; EF is either unitary or has 3 components. But either way I am not convinced that the literature currently supports a strong claim in either direction. The Miyake et al. (2000) paper was followed by a study by Miyake and Friedman (2012) demonstrating that, in adolescents and young adults, two dissociable EF components can be identified, as well as a 'common EF' component that is shared across tasks (and it should be noted, that this model is specific to the tasks used; the authors acknowledge that different tasks might yield different conclusions). Amongst pre-schoolers it has been argued that a single latent EF construct best describes pre-schoolers' performance on EF batteries (Hughes, Ensor, Wilson, & Graham, 2009; Senn, Espy, & Kaufmann, 2004; Wiebe, Espy, & Charak, 2008; Wiebe et al., 2011; Willoughby, Wirth, Blair & Family Life Project Investigators, 2012), and that EF factors can be dissociated (Bernier, Carlson, Deschênes, & Matte-Gagné, 2012; Garon, Smith, & Bryson, 2014; Mulder, Hoofs, Verhagen, van der Veen, & Leseman, 2014; Skogan et al., 2015).

**We agree that there is no clear answer on how to best separate and explain the components of executive function as factors analysis with different age groups yielded mixed findings. In our opinion, Miyake's influential work with adults identified three core factors within the central executive function, inhibition, working memory and shifting and such categorisation has become the backbone of the majority of developmental studies. We think the most appropriate way to view executive function in early childhood is to consider it as a unitary construct but with partially dissociable components (Hughes, Ensor, Wilson, & Graham, 2009; Garon, Bryson & Smith, 2008; Miyake et al., 2000; Miyake & Freeman, 2012; Wiebe, Espy, & Charak, 2008). In addition, there are wide variations in children 's performance on a range of age-sensitive tasks (Carlson, 2005). Therefore, we selected four well-established tasks for our study. We have added a paragraph to the introduction and discussion to clarify this point (line 110-126, 409-414).**

13. Line 393: It should be clarified that in this study one 1 inhibition task correlated with flexible planning performance

**Due to changes in the analysis our results have changed, therefore this section has been rewritten to represent these new results.**

**Reviewer: 2**

Comments to the Author(s)
Comments to the Author

This is my first review of the manuscript entitled "Executive function, language and flexible planning in young children."
This cross-sectional research builds on a new experimental tool-use paradigm designed to assess flexible planning in 3 to 5 year olds. The task is designed to address particular flaws that have been observed in earlier studies on this topic. Particularly, single trials were used to avoid repeated exposure to stimulus-reward relationships, training trials provided the participants with experience of a predictable return of reward, children were not verbally cued during the task, and delays were included before and after tool choice. Relations between flexible planning performance in this paradigm and receptive language as well as executive functioning were also examined. See comments to authors below.

Title: My understanding is that the flexible planning is the main part of this study and therefore I think it would be more descriptive with a title like, Flexible planning in 3-5 year old children and relations

**We have changed the title to better represent the focus of this study on flexible planning.**

Introduction
The introduction is relatively well-written, but would gain from a re-write/structure in relation to the order of the aims of this study, which appears to be several and somewhat unclear and unstructured. I would recommend the authors to think through what the main, second and third aim etc of this study really are and structure the introduction in the same order. To my understanding the following aspects are included in aims: to design a task that can be comparable with non-human studies, to design a task that addresses flaws in earlier paradigms on the same topic ( see paragraph above), to examine the role of delay of gratification in flexible planning, to examine relations with language and several executive functions. Please order these according to their significance and re-structure the introduction correspondingly.

**The introduction has been restructured to better clarify our aims and their respective backgrounds.**

Surprisingly, there are no predictions/ hypotheses formulated regarding the research questions asked. Please add predictions in relation to each research questions under the aims section.

**Predictions have been added to the aims section (line 100-103, 157-159).**

A minor (yet important) point in the introduction ( and discussion) is that the authors describe executive functions as a unitary function. As is empirically established by Miyake and others ( who the authors also refer to) EF is a complex and very broad higher order cognitive construct that show both unity and diversity and should be referred to accordingly. Please change.

**We have changed our description of executive functions and their characterisation in the introduction (line 110-126) and discussion (line 409-414).**

Methods

Subjects. The sample should be described more thoroughly. What was the inclusion/exclusion criteria? Were they all typically developing? Born after week 36? Etc. Measures of SES should be included ( for example; income and parents 'education level).

**The reviewer raises a good point that should be considered in future studies. However, we did not collect any data on parents' income levels, education, gestational age at birth etc.**

Apparatus. Although the supplementary movie and figures were helpful in understanding the apparatus and procedure. Indeed, the apparatus and procedure used is quite complex and the authors need to make sure that it is clearly described in the manuscript to ease the understanding of the readers. For example, make sure to describe the three different apparatuses used in the same or that they are illustrated in Fig 1. Or if described in the same order as they were used, please change order of picture accordingly. I had a hard time understanding the apparatus and the different steps in the procedure and had to read these sections many times. Please think through how this section can be described even more clearly, so the reader can understand it without having to watch the movie.

**Letters were added to Figure 1 and the description of the apparatuses has been modified (line 181-188) in the hope that this is now transparent. The procedure section was clarified, including clearer references to the different setups in Figure 2 (line 132-140).**

Procedure

On p.7 the authors describe that the interval between sessions depended on school arrangement and availability of subjects and that it could vary between 1 hour and 2 days. This large variation is not further discussed. Could this large interval variation have had an effect on the result? The authors write that it was typically around 2 hours. I believe a more precise measure of interval variation ( M and SD) would be meaningful in order evaluate its possible effect .

**There were 7 children (out of 87) who received the test session on a different day than the training session. Due to this small number, it was not possible to determine whether this had an influence on their performance. We have now clarified this in the "Procedure" section (line 200-202).**

Also, the testing seem to have been conducted by three different experimental leaders (RM AF and ND). Did the authors test for a possible test leader effect? If not, Please check this.

**The three researchers followed a strict script and often worked in pairs to ensure that the procedure was identical for all researchers. In those cases, one researcher would quietly sit in the room and not interfere, while the other researcher was working with the subject. We did not want to include more fixed effects than necessary in our GLMMs, therefore we did not test for this effect.**

Data Analysis and Results

No information is given regarding outliers, normality tests etc. Please add.A very important point is that no descriptive data (M , SD; min, max for the different age groups for all of the

tasks) is provided neither in the manuscript nor in the supplementary analysis. Such data is key for interpreting the results especially regarding the executive function measures and their relation to the flexible planning task. For example, were there any floor or ceiling effects for the EF tasks? For example, there were no age effects on the knock-tap task. Could it be that this lack of age effect is due to floor or ceiling effects in these children? Please add descriptive data for all the tasks included in the study.

**A table indicating the mean performance ± standard deviation for each test and age group was added (Table 3). The data presented in this table shows that there were no ceiling effects for any of the EF tasks, and that mean performance improved with age for all EF tasks, though not always significantly, except the forward digit tasks, where 4 and 5-year olds performed similarly.**

Discussion

Overall, from my perspective, the take home message of this study is not clearly conveyed in the discussion. What is really the key finding from this study? And how does it contribute novel knowledge to the field. Please state this essential information clearly in the beginning as well as at the end of the discussion ( as well as in the abstract). Similarly, to the introduction, I think that the discussion would gain from reorganizing/restructuring in relation to aims and key findings. In its current form, the reasoning is hard to follow and gives a jumbled impression.

**The discussion has been restructured and our main results have been summarised better, to clarify the take home message of this study.**

Here are some other points that I am not clear about in the discussion/ interpretation of the results.

To me, it was surprising that the EF measures were significantly correlated with one another ( please remove the word “highly” from this sentence (p.13) as the effect size of these correlations cannot be considered as strong/high (Ref Cohen ) as this is certainly not always the case with young children.

**This sentence was changed and the word “highly” was removed.**

Considering that EF is a non-unitary construct with different developmental trajectories for the different components, I think it would have been interesting to see how the EF tasks related to each other within the three age groups.

**We present correlations between success on the flexible planning task, and the executive function and language ability asks, for each age group in the supplementary material (Supplementary table 5).**

It was interesting to note that the only EF measure that correlated with the flexible planning task was the knock tap task, which was also the only EF measure that did not show age effects. How does this fact influence the interpretation of the result? Again, here it is crucial to know the descriptive data in order to make a more complete interpretation of the observed relation between these measures. Since the knock and tap task taxes the same type of complex inhibition as the day/night task ( keep a rule in mind as well as inhibiting the most

spontaneous response in favor of the correct response) except for the verbal component in the day/night task, makes me wonder what this relation really stands for. It may be non-executive, perhaps motoric, in nature. The authors do touch upon this reasoning, but I think it could be spelled out more clearly.

**Table 3 was added to the manuscript to give a better idea of the actual performance in each task. As we had to rerun our analysis, some of the correlations have changed and the results and discussion sections were altered to reflect these results. The knock-tap task does not longer correlate with the flexible planning task.**

Having said this, I am not really sure what the authors mean by saying that the lack of correlation with our other inhibitory controls measure ( day/night task) indicate that there does not appear to be a "general difficulty" with inhibition influencing flexible planning performance. As mentioned above, both inhibitory tasks measure the same type of complex inhibition and one but not the other related to the planning task. Thus, I do not believe that this conclusion can be drawn based on the data.

**We agree with your comments and have removed this sentence.**

Also, the reasoning around the relations between executive function and flexible planning on p.14 appears quite weak, e.g., "Inhibition may correlate with performance in some flexible planning task" –please elaborate on type of inhibition and what aspect of the planning that is referred to.

**As none of the executive function tasks correlate with the flexible planning task after reanalysing the data, this section has been removed.**

Since the correlational data is not included in the manuscript, it is important that the direction of the relationships are clearly expressed in the discussion. Please add this information.

**This data was now added in Table 4.**

Limitations of this study should be stated. Please add (e.g., the study being cross-sectional). No measure of episodic memory.

**A section on limitations has been added to the discussion (line 282-287).**

Finally, the task is suggested to be of use for research on this topic in other species. I am not convinced that this would actually work (what species did the authors have in mind more precisely). Please elaborate.

**This same paradigm, using almost identical apparatuses and tools, has already been used in a study on New Caledonian crows (currently submitted for publication). We have clarified what species we had in mind (line 387).**

# Appendix B

Dear Dr Dunn,

Thank you very much for accepting our manuscript ID RSOS-192015 entitled "A novel test of flexible planning in relation to executive function and language in young children" for publication subject to minor revisions. We have now made the minor revisions requested and attach the following response to comments. Please note that line numbers correspond to the tracked changes version of the manuscript.

Yours Sincerely,

Rachael Miller, Anna Frohnwieser, Ning Ding, Camille Troisi, Martina Schiestl, Romana Gruber, Alex Taylor, Sarah Jelbert, Markus Boeckle and Nicky Clayton

**Response to Editor and reviewer comments:**

Dear Dr Frohnwieser

On behalf of the Editor, I am pleased to inform you that your Manuscript RSOS-192015 entitled "Flexible planning, executive function and language in young children" has been accepted for publication in Royal Society Open Science subject to minor revision in accordance with the referee suggestions.  Please find the referees' comments at the end of this email.

The reviewers and Subject Editor have recommended publication, but also suggest some minor revisions to your manuscript. Therefore, I invite you to respond to the comments and revise your manuscript.

• Ethics statement
If your study uses humans or animals please include details of the ethical approval received, including the name of the committee that granted approval. For human studies please also detail whether informed consent was obtained. For field studies on animals please include details of all permissions, licences and/or approvals granted to carry out the fieldwork.

**Done**

• Data accessibility
It is a condition of publication that all supporting data are made available either as supplementary information or preferably in a suitable permanent repository. The data accessibility section should state where the article's supporting data can be accessed. This section should also include details, where possible of where to access other relevant research materials such as statistical tools, protocols, software etc can be accessed. If the data has been deposited in an external repository this section should list the database, accession number and link to the DOI for all data from the article that has been made publicly available. Data sets that have been deposited in an external repository and have a DOI should also be appropriately cited in the manuscript and included in the reference list.

If you wish to submit your supporting data or code to Dryad (http://datadryad.org/), or modify your current submission to dryad, please use the following link: http://datadryad.org/submit?journalID=RSOS&manu=RSOS-192015

**Done**

• Competing interests
Please declare any financial or non-financial competing interests, or state that you have no competing interests.

**Done**

• Authors’ contributions
All submissions, other than those with a single author, must include an Authors’ Contributions section which individually lists the specific contribution of each author. The list of Authors should meet all of the following criteria; 1) substantial contributions to conception and design, or acquisition of data, or analysis and interpretation of data; 2) drafting the article or revising it critically for important intellectual content; and 3) final approval of the version to be published.

All contributors who do not meet all of these criteria should be included in the acknowledgements.

We suggest the following format:
AB carried out the molecular lab work, participated in data analysis, carried out sequence alignments, participated in the design of the study and drafted the manuscript; CD carried out the statistical analyses; EF collected field data; GH conceived of the study, designed the study, coordinated the study and helped draft the manuscript. All authors gave final approval for publication.

**Done**

• Acknowledgements
Please acknowledge anyone who contributed to the study but did not meet the authorship criteria.

**Done**

• Funding statement
Please list the source of funding for each author.

**Done**

Please note that we cannot publish your manuscript without these end statements included. We have included a screenshot example of the end statements for reference. If you feel that a given heading is not relevant to your paper, please nevertheless include the heading and explicitly state that it is not relevant to your work.

Because the schedule for publication is very tight, it is a condition of publication that you submit the revised version of your manuscript before 01-Mar-2020. Please note that the revision deadline will expire at 00.00am on this date. If you do not think you will be able to meet this date please let me know immediately.

To revise your manuscript, log into https://mc.manuscriptcentral.com/rsos and enter your Author Centre, where you will find your manuscript title listed under "Manuscripts with Decisions". Under "Actions," click on "Create a Revision." You will be unable to make your revisions on the originally submitted version of the manuscript. Instead, revise your manuscript and **upload a new version** through your Author Centre.

When submitting your revised manuscript, you will be able to respond to the comments made by the referees and upload a file "Response to Referees" in "Section 6 - File Upload". You can use this to document any changes you make to the original manuscript. In order to expedite the processing of the revised manuscript, please be as specific as possible in your response to the referees.

When uploading your revised files please make sure that you have:

1) A **text file** of the manuscript (tex, txt, rtf, docx or doc), references, tables (including captions) and figure captions. Do not upload a PDF as your "Main Document".
2) A separate electronic file of each **figure** (EPS or print-quality PDF preferred (either format should be produced directly from original creation package), or original software format)
3) Included a **100 word media summary** of your paper when requested at submission. Please ensure you have entered correct contact details (email, institution and telephone) in your user account - **done**
4) Included the **raw data** to support the claims made in your paper. You can either include your data as electronic supplementary material or upload to a repository and include the relevant doi within your manuscript - **done**
5) All supplementary materials accompanying an accepted article will be treated as in their final form. Note that the Royal Society will neither edit nor typeset supplementary material and it will be hosted as provided. Please ensure that the supplementary material includes the paper details where possible (authors, article title, journal name).

Supplementary files will be published alongside the paper on the journal website and posted on the online figshare repository (https://figshare.com). The heading and legend provided for each supplementary file during the submission process will be used to create the figshare page, so please ensure these are accurate and informative so that your files can be found in searches. Files on figshare will be made available approximately one week before the accompanying article so that the supplementary material can be attributed a unique DOI.

Once again, thank you for submitting your manuscript to Royal Society Open Science and I look forward to receiving your revision. If you have any questions at all, please do not hesitate to get in touch.

Kind regards,
Andrew Dunn
Royal Society Open Science Editorial Office
Royal Society Open Science
openscience@royalsociety.org

on behalf of Dr Teodora Gliga (Associate Editor) and Essi Viding (Subject Editor)
openscience@royalsociety.org

Associate Editor Comments to Author (Dr Teodora Gliga):

Associate Editor
Comments to the Author:
Your revision was now seen by the two original reviewers. Although they both indicate that you have addressed many of their comments, there remain some inconsistencies in the way you describe the literature or the findings, that you need to address. Most importantly, I urge you to clearly state the **aims of the study** and make sure these are consistently indicated throughout the manuscript (title, abstract, main text).

**We have now revised the manuscript and supplementary materials in accordance with the Editor and reviewer comments – in particular, as suggested, we've clarified the main aims of the study through-out the ms.**

**Reviewer comments to Author:**
**Reviewer: 1**

Comments to the Author(s)
The authors have responded appropriately to the previous round of comments and this manuscript provides a well-reasoned explanation of the study design and results. I have some further minor amends to suggest, below, but no major concerns with the manuscript as it currently stands.

**Thank you.**

Minor amends
1. Given that the authors concede that the tool is gratifying so the sticker-tool choice doesn't actually work as a delay of gratification task, the sentence
First, we tested delay of gratification by asking children to choose between an immediately available reward of lower quality or a tool to obtain a delayed reward of higher quality
Should be amended to
First, we aimed to test delay of gratification by asking children to choose between an immediately available reward of lower quality or a tool to obtain a delayed reward of higher quality

**We now changed the sentence to "we aimed to test delay of gratification" (line 104)**

2. Add to the limitations an acknowledgement that this (presumably) was not a diverse sample, drawn only from a British sample and with no monitoring or attempt to diversity SES

**We now acknowledge these limitations of our sample, which was drawn from children in UK with not monitoring or attempt to diversify SES (line 413)**

3. Add to the limitations section that 7 out of 87 children were tested on a different day to the training session

**We added now that 7 out of the 87 children were tested on a different day for the training session (line 414)**

4. Add to the Methods section that participants are presumed to be typically-developing but that no screening was attempted

**We have now added that we presumed the children to be typically developed (line 186-187)**

5. In the sentence “Adopting factorial analytic approach, researchers have identified three key components of executive factors; inhibitory control, working memory and cognitive flexibility” clarify that this is in adults

**We now clarified this sentence by adding “in adults” (line 126)**

6. The sentence “Executive function is a unitary construct with three core components: inhibition, working memory and cognitive shifting/flexibility” is not justified by the arguments above. It is possible, on the basis of the 2 studies mentioned, that the nature of executive function changes across development, from a primarily inhibitory (‘Common EF’) function, to a more complex, multi-component function in later development. Further there are other influential theoretical models of EF and the Miyake model only strictly applies to the population and tasks used in that study. To allow for other models, the framing should be something like ‘Influenced by the work of Miyake et al which showed that ….. along with developmental research indicating that in young children EF may be a more unitary construct driven primarily by inhibitory control… we have selected…..”

**Edited as suggested: “One model, influenced by the work of Miyake et al, along with developmental research, indicates that in young children, executive function may be a more unitary construct comprising inhibition, working memory and cognitive shifting/flexibility - though driven primarily by inhibitory control” (lines 126-130) and also in the discussion (lines 442-445)**

7. Clarify the expected direction of the correlations (last sentence before the methods)

**We now clarified the expected direction of the correlation (line171, 176)**

8. Add details of the EF task administration protocols in the Supplementary materials, and references to the original sources

**We’ve added this information to the Supplementary Materials as suggested. We do also already reference the original sources in Table 2 (references).**

9. In the methods section, indicate which EF task is designed to index which component skill

**Table 2 provides this information – but we’ve added it into the text briefly as well as suggested (line 280-282). Inhibition = knock-tap, day-night, cognitive flexibility = dimensional change card sort (DCCS), working memory = forward and backward digit span, receptive language = British Picture Vocabulary Scale**

10. The following sentence seems incomplete “For example, corvids (members of the crow family) have shown impressive flexible planning skills in a caching, for example in learning what to cache and what not to cache based on the foods available at the time of cache recovery “

**We have completed the sentence "For example, corvids (members of the crow family) have shown impressive flexible planning skills in caching tasks, for example in learning what to cache and what not to cache based on the food available at the time of cache recovery" (lines 78)**

11. Should Subjects not be participants throughout?

**We now have changed "subjects" to "participants" throughout the manuscript**

12. It is not clear to me that the logic of the following sentence holds true: "Finally, as the children performed similarly in our flexible planning task as in previous developmental studies, we feel our new paradigm provides an exciting opportunity to further explore episodic foresight across different species, specifically corvids, such as tool-using Caledonian crows, or primates". Why should showing a developmental improvement in children mean the task could be used in non-human animals? Please make this argument clearer, or drop. This point also applies to the very last sentence.

**We have now expanded on this argument as part of clarifying the aims of this study as suggested by reviewer 2 and the Editor.**

**Reviewer: 2**

Comments to the Author(s)
The authors have done a pretty good job with addressing most of my comments in the previous version of this manuscript.

**Thank you.**

However, I am quite concerned about the fact that the main/or overall aim of this study is still not clear. Is it to **validate this new task of flexible planning by looking at expected age effects** and **in addition investigating relations to executive function performance and language as part of this validation**? In the aim section at the end of the introduction the main aim is described as "just" studying correlations between this new task and executive functions and language, whereas in the discussion it appears as the relations between different functions is a secondary aim. Further, the title says nothing about that **a novel task of flexible planning** is explored. Again, I think the authors really need to think through what the main aim really is and the clearly and consistently express this throughout the manuscript.

**Yes, that was our aim, which we have now clarified in the abstract, intro and discussion. We have also changed the title again to reflect that this is a novel test, as suggested.**

Also, the sentence now describing the main aim (line 248) begins by saying Our third aim – please delete third here.

Further, analyses for the relations between flexible planning and executive functions and language are provided per age group, but predictions are only included for the total sample regarding these relations. Do the authors expect age effects in these relations? Please add predictions regarding the relations per age group.

**We now deleted “third” from “our third aim” and added in predictions within age groups (line 176).**

Minor: it appears that executive function on p.17 is still described as unitary construct. If the authors mean that previous findings show that the construct seem to load on a single factor in early development and in the preschool year this should be more clearly expressed.

**Added this as suggested to line 136**

Also there seem to be reiteration between the sentence on line 183-187 and the one on line 192-198. I suggest the last part of the first sentence ( 183-187) -"yet preschoolers performance on various tasks can be explained by a single theoretical latent factor" should be deleted. By the way, it is unclear to me what a theoretical factor here means.

**We now have deleted “yet preschoolers’ performance on various tasks can be explained by a single theoretical latent factor”.**
